# Comprehensive analysis of the aldehyde dehydrogenase gene family in *Phaseolus vulgaris* L. and their response to saline–alkali stress

**DOI:** 10.3389/fpls.2024.1283845

**Published:** 2024-02-21

**Authors:** Xiaoqin Wang, Mingxu Wu, Song Yu, Lingxia Zhai, Xuetian Zhu, Lihe Yu, Yifei Zhang

**Affiliations:** ^1^ College of Agriculture, Heilongjiang Bayi Agricultural University/Heilongjiang Provincial Key Laboratory of Modern Agricultural Cultivation and Crop Germplasm Improvement, Daqing, Heilongjiang, China; ^2^ Keshan Branch of Heilongjiang Academy of Agricultural Sciences, Keshan, Heilongjiang, China; ^3^ Key Laboratory of Low-carbon Green Agriculture in Northeastern China, Ministry of Agriculture and Rural Affairs, Daqing, Heilongjiang, China

**Keywords:** common bean, aldehyde dehydrogenase, abiotic stress, saline-alkali stress, expression pattern

## Abstract

**Background:**

Aldehyde dehydrogenase (ALDH) scavenges toxic aldehyde molecules by catalyzing the oxidation of aldehydes to carboxylic acids. Although ALDH gene family members in various plants have been extensively studied and were found to regulate plant response to abiotic stress, reports on *ALDH* genes in the common bean (*Phaseolus vulgaris* L.) are limited. In this study, we aimed to investigate the effects of neutral (NS) and basic alkaline (AS) stresses on growth, physiological and biochemical indices, and ALDH activity, *ALDH* gene expression of common bean. In addition, We used bioinformatics techniques to analyze the physical and chemical properties, phylogenetic relationships, gene replication, collinearity, cis-acting elements, gene structure, motifs, and protein structural characteristics of PvALDH family members.

**Results:**

We found that both NS and AS stresses weakened the photosynthetic performance of the leaves, induced oxidative stress, inhibited common bean growth, and enhanced the antioxidative system to scavenge reactive oxygen species. Furthermore, we our findings revealed that ALDH in the common bean actively responds to NS or AS stress by inducing the expression of *PvALDH* genes. In addition, using the established classification criteria and phylogenetic analysis, 27 *PvALDHs* were identified in the common bean genome, belonging to 10 ALDH families. The primary expansion mode of *PvALDH* genes was segmental duplication. Cis-acting elemental analysis showed that *PvALDHs* were associated with abiotic stress and phytohormonal responses. Gene expression analysis revealed that the *PvALDH* gene expression was tissue-specific. For instance, *PvALDH3F1* and *PvALDH3H1* were highly expressed in flower buds and flowers, respectively, whereas *PvALDH3H2* and *PvALDH2B4* were highly expressed in green mature pods and young pods, respectively. *PvALDH22A1* and *PvALDH11A2* were highly expressed in leaves and young trifoliates, respectively; *PvALDH18B2* and *PvALDH18B3* were highly expressed in stems and nodules, respectively; and *PvALDH2C2* and *PvALDH2C3* were highly expressed in the roots. *PvALDHs* expression in the roots responded positively to NS–AS stress, and *PvALDH2C3*, *PvALDH5F1*, and *PvALDH10A1* were significantly (*P* < 0.05) upregulated in the roots.

**Conclusion:**

These results indicate that AS stress causes higher levels of oxidative damage than NS stress, resulting in weaker photosynthetic performance and more significant inhibition of common bean growth. The influence of *PvALDHs* potentially modulates abiotic stress response, particularly in the context of saline–alkali stress. These findings establish a basis for future research into the potential roles of *ALDHs* in the common bean.

## 1 Introduction

Common bean (*Phaseolus vulgaris* L) is an annual herbaceous plant belonging to the family Leguminosae and subfamily Papilionoideae. As an essential leguminous plant, the common bean is rich in nutrients, such as proteins, fiber, carbohydrates, minerals, and vitamins (Duc et al., 2015; Torche et al., 2018). In addition, the common bean contains high levels of polyphenol compounds, which are crucial to human health by preventing cardiovascular disease, hyperglycemia, obesity, and colon cancer (Chung et al., 2008; Hayat et al., 2014). Recently, the gradual improvements in living standards and the ongoing refinement of consumption patterns have resulted in annual growth in the global demand for common beans. However, various abiotic and biological stresses often affect the yield and quality of common beans (Beebe et al., 2013; Yu et al., 2019).

Aldehyde molecules are common intermediates produced by catabolic and biosynthetic pathways during biological growth and development, and they can be formed through various metabolic processes, such as amino acid, carbohydrate, lipid, bioamine, vitamin, and steroid metabolism (Vasiliou et al., 2000). During their life cycle, plants frequently encounter a range of abiotic and biological challenges, including drought, salinity, cold temperatures, and diseases (Suzuki et al., 2014). In response to these stress hazards, plants produce numerous reactive oxygen species (ROS) and toxic compounds, accumulating intracellular aldehydes. Although aldehydes are indispensable substances in organisms, excessive aldehyde accumulation interferes with the metabolic balance and induces toxic conditions (Kirch et al., 2005; Stiti et al., 2011). Therefore, the balance of intracellular aldehyde content must be controlled to facilitate the optimal growth and development of living organisms. Aldehyde dehydrogenase (ALDH, EC: 1.2.1.3) consists of various NAD(P)-dependent enzymes that can irreversibly oxidize various aromatic and aliphatic aldehydes into their respective carboxylic acids, effectively clearing excess aldehydes in the body and maintaining stable intracellular metabolism (Yoshiba et al., 1997; Yoshida et al., 1998). Moreover, ALDHs are essential for maintaining redox homeostasis of plant cells by changing the content of NAD(P)H and the total amount of glutathione (Missihoun et al., 2018). Furthermore, earlier research has established that ALDH plays an important role in a multitude of physiological processes, such as amino acid metabolism, gluconeogenesis, glycolysis, and carnitine biosynthesis (Marchitti et al., 2008; Tylichová et al., 2010; Yang et al., 2011).

ALDHs are ubiquitous, occurring in both eukaryotic and prokaryotic organisms, and they exhibit robust representation across nearly all plant species (Brocker et al., 2013). To date, 24 ALDH families (ALDH1–ALDH24) have been identified in accordance with the ALDH Gene Nomenclature Committee’s criteria (Islam et al., 2022). Fourteen different ALDH families have been found in plants (ALDH-2, 3, 5, 6, 7, 10, 11, 12, 18, 19, 21, 22, 23, and 24), among which seven families are unique to plants (ALDH-11, 12, 19, 21, 22, 23, and 24) (Zhang et al., 2012). Currently, the presence of ALDH19 has exclusively been detected in Solanum lycopersicum. In this species, it encodes γ-glutamyl phosphate reductase, a crucial enzyme involved in the synthesis of proline from glutamate (García-Ríos et al., 1997).

Continuous research development has led to the discovery of an increasing number of *ALDHs* and their potential roles in plants. For example, *ALDH3I1* and *ALDH7B4* in Arabidopsis thaliana can enhance salt and drought tolerance by reducing the accumulation of H_2_O_2_ and malondialdehyde (MDA) caused by lipid peroxidation in cells (Kotchoni et al., 2006). Overexpression of *BrALDH7B2* in tobacco can improve plant salt tolerance by enhancing photosynthetic performance in leaves, reducing the production of ROS, and inhibiting cell death in roots (Gautam et al., 2020). *ALDH2C4* inhibition in *Nicotiana benthamiana* led to increased sensitivity to low-temperature stress and significant increases in MDA and ROS (Guo J. et al., 2020). Following the overexpression of the *VvALDH2B4* of Chinese wild grapevine (*Vitis pseudoreticulata*) in *Arabidopsis*, the MDA levels of the transgenic lines decreased, and their resistance to salt stress and pathogenic bacteria increased (Wen et al., 2012).

The Songnen Plain in Northeast China is an important production area for common beans because of its flat terrain and fertile soil. However, owing to global climate change, secondary salinization has been increasing in this region yearly, hindering the productivity and quality of common beans (Talaat, 2014; Yu et al., 2019). Therefore, studying the adaptation mechanism of common beans to abiotic stress, especially saline–alkali stress, would improve the tolerance of common beans to field environmental stress. Moreover, it would promote the yield and quality of common beans and facilitate the efficient development of common beans in this area. Although the *ALDH* gene family members in numerous plants have been widely studied and their potential contribution to plant response under abiotic stress has been confirmed, reports on the *ALDH* genes in the common bean are relatively scarce.

In the present study, we explored the response of common bean growth and physiological indicators to neutral (NS) and alkaline salt (AS) stresses. Moreover the whole genome of the ALDH family of common beans was identified. The analysis of the physicochemical properties, phylogenetic relationships, gene replication, collinearity, cis-acting elements (CAEs), gene structure, motif, protein structure, and expression patterns of members of the ALDH family in common beans highlights the potential of the *ALDH* gene family in the response of common beans to abiotic stress.

## 2 Materials and methods

### 2.1 Plant materials and NS–AS stress treatment

Using “Qingyun No. 1”, a high-yield and high-resistance common bean variety in Northeast China, as the tested material, the selected high-quality seeds were disinfected and planted in a pot (material: polyvinyl chloride, diameter: 12 cm, height:11 cm) filled with soil mixture (black soil:vermiculite = 1:1). Four holes were made evenly in each pot, and two seeds were planted in each hole, while only one young plant was left in each hole after emergence. The weighing method was used to replenish water daily to maintain the soil water capacity at 70%. When the first compound leaf was fully unfolded, the plants were treated with 250 mM NaCl solution (NS stress) or 250 mM Na_2_CO_3_ and NaHCO_3_ mixed in a 1:9 molar ratio solution (AS stress). Each basin was irrigated with 200 mL of the treatment solution, and the same volume of distilled water was used as the control treatment.

### 2.2 Determination of growth and physiological indicators

After 72 h of NS or AS stress treatment, the stomatal conductance (*Gs*), net photosynthetic rate (*Pn*), transpiration rate (*Tr*), and intercellular CO_2_ concentration (*Ci*) of the first completely unfolded triple leaves were examined using an LI-6400 portable photosynthetic device (Li Cor BioScience, Lincoln, NE, USA). Subsequently, a portion of the tested plants was used to assess physiological and biochemical indicators and related gene expression. Furthermore, the root and shoot samples of the remaining test plants were separated and dried in an electric blast drying oven (BGZ-306, Shanghai Boxun Medical Biological Instrument Co., LTD, Shanghai, China) at 105°C for 30 min, and further dried at 85°C until the weight remained constant to determine the dry weight. The SPECORD Plus 210 UV-Vis spectrophotometer (Analytik Jena, Germany) was employed to measure the levels of MDA, chlorophyll, H_2_O_2_, superoxide radical (O_2_^•-^), superoxide dismutase (SOD), peroxidase (POD), and catalase (CAT).The amount of chlorophyll was also determined using Sartory and Grobbelaar (1984) methodology.

SOD, POD, and CAT enzymes were extracted according to the method proposed by De Azevedo Neto et al. (2005). First, 0.5 g of leaf or root tissue samples was homogenized in cold extraction buffer containing 5 mL of 50 mM sodium phosphate buffer (pH 7.8 for SOD and POD; pH 7.0 for CAT), 0.1 mM Na2EDTA, and 1% (w/v) polyvinylpyrrolidone. Then, the homogenate was centrifuged at 12000 g for 20 min at 4°C, and the resulting supernatant was used as the extract for testing. SOD activity was determined according to the nitrogen blue tetrazolium method described by Giannopolitis and Ries (1977). POD activity was determined according to the guaiacol method described by Chance and Maehly (1955). CAT activity was determined according to the method described by Havir and McHale (1987).

The hydrogen peroxide (H_2_O_2_) content was determined using the trichloroacetic acid (TCA) method described by Velikova et al. (2000). Approximately 0.5 g leaf or root tissue was mixed with 5 mL 0.1% (w/v) TCA ice bath homogenate. Subsequently, the supernatant obtained by from 12 000 g centrifugation for 15 min was used to determine the content of H_2_O_2_.

The hydroxylamine oxidation method described by Elstner et al. (1975) was used to determine O_2_^•-^ content. Approximately 1.0 g of leaf or root tissue was added to 3 mL of 65 mM potassium phosphate buffer (pH 7.8) ice bath homogenate. Then, the supernatant obtained after centrifugation at 10000 g for 3 min was used to determine O_2_^•-^ content.

MDA content was determined using the thiobarbituric acid reaction according to the method described by Hodges et al. (1999). Leaf or root samples (0.5 g) was homogenized in 5 mL 0.1% (w/v) TCA solution. The supernatant obtained by centrifugation at 10000 g for 20 min was used for the determination of MDA content.

### 2.3 ALDH enzyme activity assay

ALDH enzyme activity was determined in root samples after 0, 24, 48, and 72 h of neutral or AS treatment. An ALDH assay kit (G0828F, Grace Biotechnology Co., Ltd, Suzhou, China) was used in which acetaldehyde is oxidized by ALDH and produces NADH, which reacts with the probe to produce a colorimetric (450 nm) product proportional to the amount of ALDH activity. The amount of enzyme catalyzing the production of 1 nmol of NADH per minute for each sample was regarded as one enzyme activity unit. According to the process described by Cao et al. (2023), approximately 0.1 g of the plant tissue sample was ground in a pre-cooled motor containing 1 mL of extraction buffer, and then, the ice-cold homogenate was centrifuged at 12000 g for 4°C. The extraction supernatant was placed on ice for measurement. The spectrophotometer was preheated for 30 min, and the wavelength was set to 450 nm. The reaction mixture was set up according to the kit instructions and mixed thoroughly in a 1-mL glass cuvette. The initial absorbance at 450 nm was measured as A1 after 30 s, and the final measurement of absorbance after 30 min was A2. Each sample was required to be its own control. Using the formula △A= (A2-A1) determination - (A2-A1) control, we calculated the change in measurement of the samples from initial to final. The standard curve was calculated as *y* = 0.0364*x*-0.0069, *R* ^2 ^= 0.9977, where *x* represents the molar mass of NADH (nmol), and y represents △A. ALDH activity was calculated as (nmol/min/g fresh weight) = [(ΔA+0.0069)/0.0364]/(sample mass×volume of added samples/addition of extraction buffer)/reaction time.

### 2.4 Identification and characterization of the *ALDHs* in the common bean

Genomic information (PhaVulg1_0) on the common bean was obtained from NCBI (https://www.ncbi.nlm.nih.gov/), and the Hidden Markov Model (HMM) file PF00171 of the ALDH conserved sequence (Finn et al., 2014) was obtained from the Pfam database (http://pfam.xfam.org/). HMMER was used to search the common bean protein database using the PF00171 model (Finn et al., 2015). Next, the identified *Arabidopsis thaliana* (TAIR10.1) ALDH protein sequence was used as a query sequence for a BLAST search (E-value < 1e^-5^). Next, the conserved domains of candidate proteins were validated using the SMART database(http://smart.embl-heidelberg.de/smart/batch), NCBI-Conserved Domain Database (http://smart.embl-heidelberg.de/smart/batch.pl), and Pfam database. In addition, the cysteine (PS00070) and glycine active sites (PS00687) of the ALDH family members were identified using Search InterPro (http://www.ebi.ac.uk/interpro/search/sequence/). Common bean ALDH family members were annotated taxonomically following the ALDH Gene Nomenclature Committee nomenclature standard (Wang et al., 2017). Accordingly, protein sequences with > 40% similarity to known ALDH sequences were classified as the same family. Those with > 60% similarity were classified as the same subfamily, and those with < 40% similarity were re-established as a novel ALDH family.

ExPASy (https://web.expasy.org/protparam/) was used to estimate protein physicochemical characteristics, including the theoretical amino acid number and isoelectric point, instability and lipid coefficients, molecular weight, and hydrophilicity. Plant-mPLoc (http://www.csbio.sjtu.edu.cn/bio-inf/plant-multi/) was used to estimate the subcellular localization of the PvALDH protein (Chou and Shen, 2010).

### 2.5 Chromosomal localization and gene duplication analysis

The chromosomal positions of the *PvALDH* genes were obtained from the NCBI database. A gene collinearity analysis was conducted with MCScanX (Wang et al., 2012), and the collinear relationships and chromosome distribution of the *ALDH* genes were visualized with Circos software (Krzywinski et al., 2009). According to the criteria of the MCScanX, a *PvALDH* gene located in an identified collinear region is considered to have been produced by whole genome or segmental duplications. Moreover, if two homologous *ALDH* genes are adjacent or separated by no more than one gene on the same chromosome, they are considered to have been generated by tandem duplication (Wang et al., 2012). The values of the synonymous rate (Ka) and non-synonymous rate (Ks) were measured using the Simple Ka/Ks Calculator program in the TBtools software (Chen et al., 2020). The ratio of Ka/Ks can reflect the selection pressure experienced during biological evolution. Ka/Ks ratios of =1, <1, and  >1 denote neutral evolution, purifying selection, and positive selection, respectively (Hasan et al., 2021). Each duplicated gene pair of PvALDH was assigned a duplication time (T) as follows: T=Ks/(2×6.1×10^−9^)×10^−6^ Mya (Lynch and Conery, 2000).

### 2.6 Gene structure, protein structural domain, and conserved motif analyses

Multiple sequence alignment and phylogenetic tree construction of PvALDH proteins were performed using MEGA X (Kumar et al., 2018). The gene structures of *PvALDHs* were determined using GSDS 2.0 (http://gsds.cbi.pku.edu.cn/). Using the MEME database (https://meme-suite.org/meme/index.html) conserved motif analyses were performed for the PvALDH family of proteins. The default parameters were utilized, while the maximum number of motifs was set to 10. The Search InterPro (http://www.ebi.ac.uk/interpro/search/sequence/) online software was used to identify the locations of ALDH-conserved domains and cysteine (PS00070) and glycine (PS00687) active sites. Mapping was performed with IBS 2.0 software (http://www.ebi.ac.uk/interpro/search/sequence/).

### 2.7 Sequence alignment and phylogenetic analysis of ALDHs

For the phylogenetic analysis of ALDH members, MEGA X was applied for multiple sequence alignments of the identified PvALDH and ALDH proteins in *Sorghum bicolor*, *Oryza sativa*, *Arabidopsis thaliana*, *Zea mays*, *Brassica rapa*, *Glycine max*, *Vitis vinifera*, *Malus domestic*, *Solanum tuberosum*, *Solanum lycopersicum*, *Populus trichocarpa*, *Homo sapiens*, *Chlamydomonas reinhardtii*, *Physcomitrella patens*, *Selaginella moellendorffii*, and *Ostreococcus tauri* (Thompson et al., 2002; Kumar et al., 2018). Phylogenetic trees were constructed using the neighbor-joining method. A bootstrap test with 1000 replicates was performed, and evolview (http://www.evolgenius.info/evolview/) was used to modify the phylogenetic tree (Guindon and Gascuel, 2003).

### 2.8 Putative promoter sequence analysis for cis-regulatory elements

The 1500-bp sequence upstream of the starting codon for all the *PvALDH* genes was retrieved from NCBI, and stress- or hormone-responsive CAEs were predicted using PlantCARE (http://bioinformatics.psb.ugent.be/webtools/plantcare/html/). The results were visualized using TBtools.

### 2.9 Analysis of tissue-specific expression profiles

Data on the expression levels of *PvALDHs* at different tissue sites and growth and developmental stages were acquired from Phytozome (https://phytozome-next.jgi.doe.gov/). A cluster analysis was performed, and a heatmap was constructed with TBtools.

### 2.10 Analysis of the gene expression levels under NS–AS stress

Samples were taken from the root and leaf of each plant following treatment for 0, 24, 48, and 72 h. Ten individual plants were selected as one biological replicate for each treatment, and three biological replicates were employed for each treatment. After sampling, the plants were rapidly snap-frozen with liquid nitrogen and placed in a -80°C freezer until RNA isolation.

Total RNA from the root and leaf exposed to neutral or alkaline salts at 0, 24, 48, and 72 h was extracted using the TRIzol Reagent (Invitrogen, USA). First, RNA quality was detected using the Nanodrop OoneC (Thermo Fisher Scientific, Waltham, MA, USA) and visualized on a 1% gel, followed by cDNA synthesis using the ReverTra AceTM qPCR RT Master Mix with gDNA Removal kit (TOYOBO Co., Osaka, Japan). Primer-BLAST online software (http://www.ncbi.nlm.nih.gov/tools/primer-blast/) was utilized for primer design before *PvALDHs* amplification, and ACT11 (Zhang et al., 2022) served as a reference gene (**Supplementary Table S1**). Subsequently, quantitative real-time polymerase chain reaction (qRT-PCR) was performed on a CFX96 real-time fluorescence quantitative PCR detection system (Bio-Rad Laboratories Inc., USA) with SYBR®-Green-PCR-Master-Mix (TOYOBO Co., Osaka, Japan). Each treatment had three independent biological replicates (three technical replicates per sample).The 2^−ΔΔCt^ method was used to determine the mRNA level of *ALDH* (Islam et al., 2019).

### 2.11 Structural feature analysis of PvALDH protein and homology modeling

The secondary structures of ALDH proteins from the common bean were predicted using the Self-Optimized Prediction Method with Alignment (https://npsa-prabi.ibcp.fr/cgi-bin/npsa_automat.pl?page=/NPSA/npsa_sopma.html). PvALDHs N-glycosylation sites were identified using the NetNGlyc 1.0 server (http://www.cbs.dtu.dk/services/NetNGlyc/). Next, PvALDH2B2, PvALDH2C3, PvALDH5F1, and PvALDH10A1 were chosen for homology modeling. SWISS-MODEL server (https://swissmodel.expasy.org/) was used to generate the PvALDH protein model. The model structure was verified using the PSVS server (https://montelionelab.chem.rpi.edu/PSVS/psvs2/), and Ramachandran plots were used to calculate the number of protein residues in both favorable and allowable regions.

### 2.12 Statistical analyses

Growth and physiological indicators, ALDH enzyme activity, and relative gene expression data for each treatment were analyzed using the SPSS 20.0 software for variance analysis. The samples were analyzed three times for the determination of growth and physiological indicators. Duncan’s multiple approach was employed to identify significant differences in the average values between groups at the 5% level. The data were visualized using Origin 8.0.

## 3 Results and analysis

### 3.1 Physiological response analysis of common bean to NS and AS stress treatments

**Figure 1:**
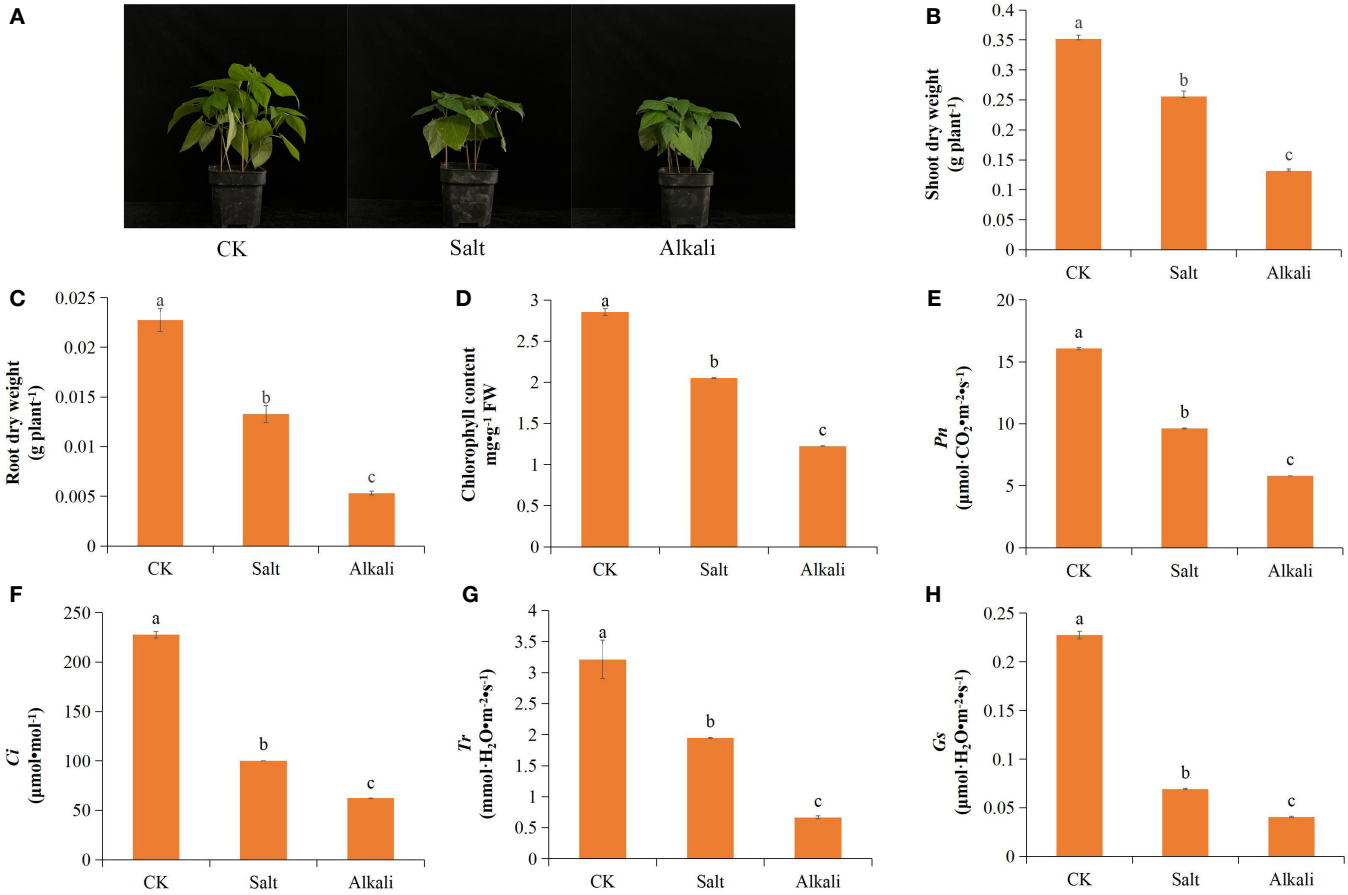
Effects of neutral and alkaline salt stresses on the growth and leaf photosynthetic performance of the common bean. **(A)** common bean phenotype; **(B)** shoot dry weight; **(C)** root dry weight; **(D)** chlorophyll content; **(E)** *Pn*; **(F)** *Gs*; **(G)** *Ci*; **(H)** *Tr*. *Pn*, net photosynthetic rate; *Gs*, stomatal conductance; *Ci*, intercellular CO_2_ concentration; *Tr*, transpiration rate; CK, control treatment. Standard deviation (SD; n = 3) is represented by error bars. Different lowercase letters indicate statistically significant differences (p < 0.05) among treatments.

**Figure 2:**
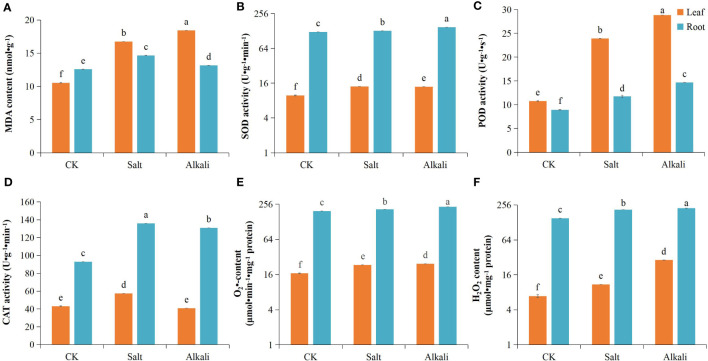
Effects of neutral and alkaline salt stresses on the oxidative stress of in common bean. **(A)** MDA content; **(B)** SOD activity; **(C)** POD activity; **(D)** CAT activity; **(E)** O_2_^•-^ content; **(F)** H_2_O_2_ content. MDA, malondialdehyde; SOD, superoxide dismutase; POD, peroxidase; CAT, catalase; O_2_^•-^: superoxide radical; H_2_O_2_, hydrogen peroxide. Orange and blue represents the common bean leaf and root, respectively. Standard deviation (SD; n = 3) is represented by error bars. Different lowercase letters indicate statistically significant differences (p < 0.05) among treatments.

NS–AS stress inhibited the growth and development of common bean plants, resulting in reduced plant height (**Figure 1A**). Compared with that in the control group, plants exposed to NS–AS stress showed significantly decreased dry weight of shoots and roots by 27.36, 62.54, 41.50, and 76.54%, at 0, 24, 48, and 72 h, respectively (**Figures 1B, C**). Both NS and AS stresses significantly (*P*<0.05) reduced *Pn*, *Gs*, *Ci*, *Tr*, and chlorophyll contents of common bean leaves compared to the control treatment, with the NS stress treatment reducing them by 40.06, 69.53, 56.01, and 39.40%, respectively, while the AS stress treatment reduced them by 63.95, 82.22, 72.66, and 79.23%, respectively (**Figures 1D–H**). In addition, compared with the control treatment, both NS and AS stresses significantly (*P* < 0.05) increased the levels of MDA, H_2_O_2_, O_2_^•-^, SOD and POD in the roots and leaves of the common bean. Under NS and AS stresses, CAT activity in the roots significantly (*P* < 0.05) increased, whereas CAT activity in the leaves only significantly increased under neutral salt stress (**Figures 2A–F**).

### 3.2 ALDH activity in common bean root under NS–AS stress

**Figure 3:**
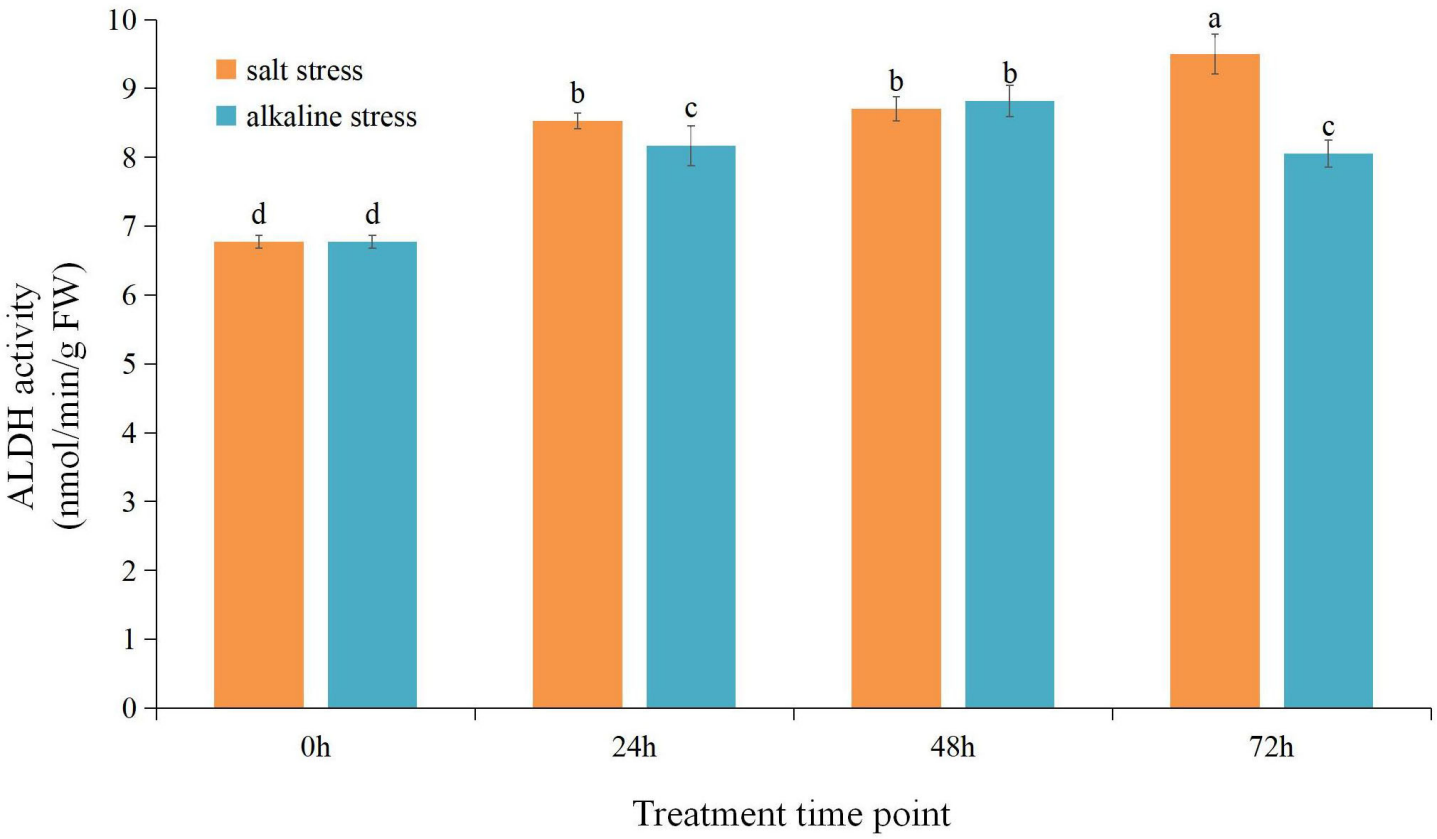
Changes in aldehyde dehydrogenase (ALDH) enzyme activity in common bean roots under neutral and alkaline salt stresses. The orange column shows ALDH enzyme activity under neutral salt stress. The blue column shows ALDH enzyme activity under the alkali salt stress. Different lowercase letters indicate statistically significant differences (p < 0.05) among treatments.

We measured the ALDH enzyme activity in the root of the common bean under different treatments. (**Figure 3**). ALDH activity significantly increased under NS–AS stress compared with that in the control group (0 h). Under NS stress, ALDH activity increased with stress time and reached the maximum value after 72 h, which was increased by 40.28%, compared with that in the control group. Under AS stress, ALDH activity initially increased and then decreased with stress time and reached the maximum value at 48 h, which was increased by 30.14%, compared with that observed in the control group.

### 3.3 Identification of the *PvALDH* gene family members

**Figure 4:**
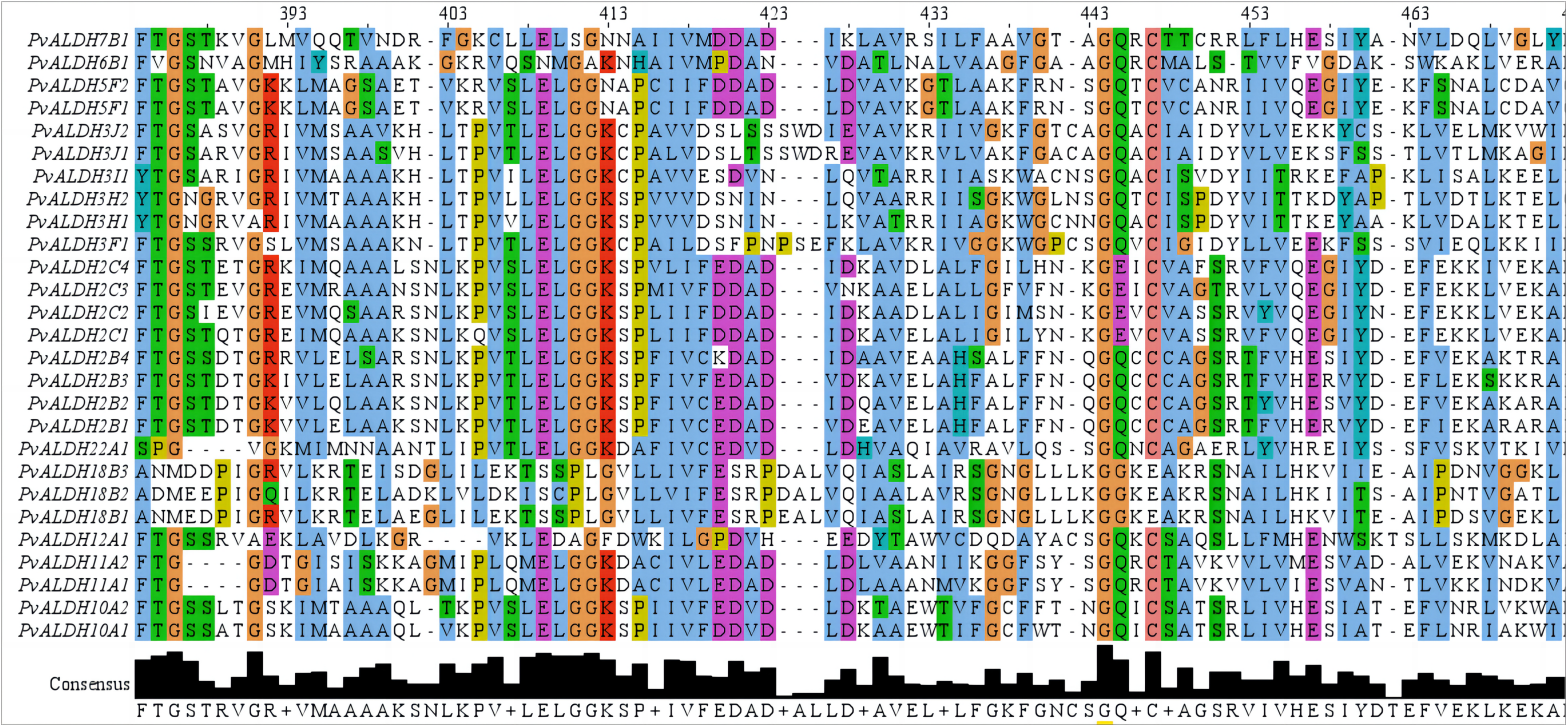
Protein domain alignment analysis of members of the PvALDH family. Editing, visualizing, and analyzing the multi-sequence alignment was carried out with JalView (https://www.jalview.org/).

We identified 27 putative ALDH family members from the common bean genome. Analysis of the NCBI Conserved Domain Database and Pfam confirmed the existence of the conserved ALDH domain (PF00171) in candidate proteins. InterPro search and multiple sequence alignment verified the occurrence of the conserved cysteine (PS00070) and glutamic (PS00687) active sites in the majority of the PvALDH proteins (**Figure 4**; **Supplementary Table S2**). A total of 14 PvALDH proteins contained both active sites. PvALDH3H1, PvALDH3H2, PvALDH2C2, and PvALDH7B1 contained only the glutamic acid active site (PS00687), whereas PvALDH6B1 and PvALDH12A1 contained only the cysteine active site (PS00070). The remaining seven proteins (PvALDH3F1, PvALDH3I1, PvALDH3J1, PvALDH3J2, PvALDH18B1, PvALDH18B2, and PvALDH18B3) had no active sites (**Figure 4**; **Supplementary Table S2**). Moreover, the catalytic glutamate residue was absent in the ALDH6 enzyme, which exhibited coenzyme A-dependent acylating activity instead. Similarly, the ALDH18 enzyme lacked the catalytic glutamate residue and demonstrated Δ-1-pyrroline-5-carboxylate synthetase activity (Brocker et al., 2013). According to the criteria set forth by the ALDH Gene Nomenclature Committee criteria, PvALDH members were classified and annotated, and all the identified PvALDH members were classified into 10 families (ALDH-2, 3, 5, 6, 7, 10, 11, 12, 18, and 22) (Brocker et al., 2013). Among these 10 families, ALDH2 had the highest number at 8 eight members; ALDH6, ALDH7, ALDH12, and ALDH22 had only one member in each family; ALDH3 had six members; ALDH18 had four members; and the remaining families had two members per family (**Supplementary Table S2**). Further analysis showed that the PvALDH proteins length varied between 347 and 720 amino acids in length, the estimated isoelectric point varied between 5.15 and 9.28, and the molecular weight varied between 23.63 and 78.12 kDa. Based on subcellular localization predictions, most PvALDH proteins were primarily localized in chloroplasts and mitochondria, followed by the cytoplasm and peroxisome (**Supplementary Table S2**).

### 3.4 Chromosomal localization, gene duplication, and collinearity analysis of *PvALDHs*

**Figure 5:**
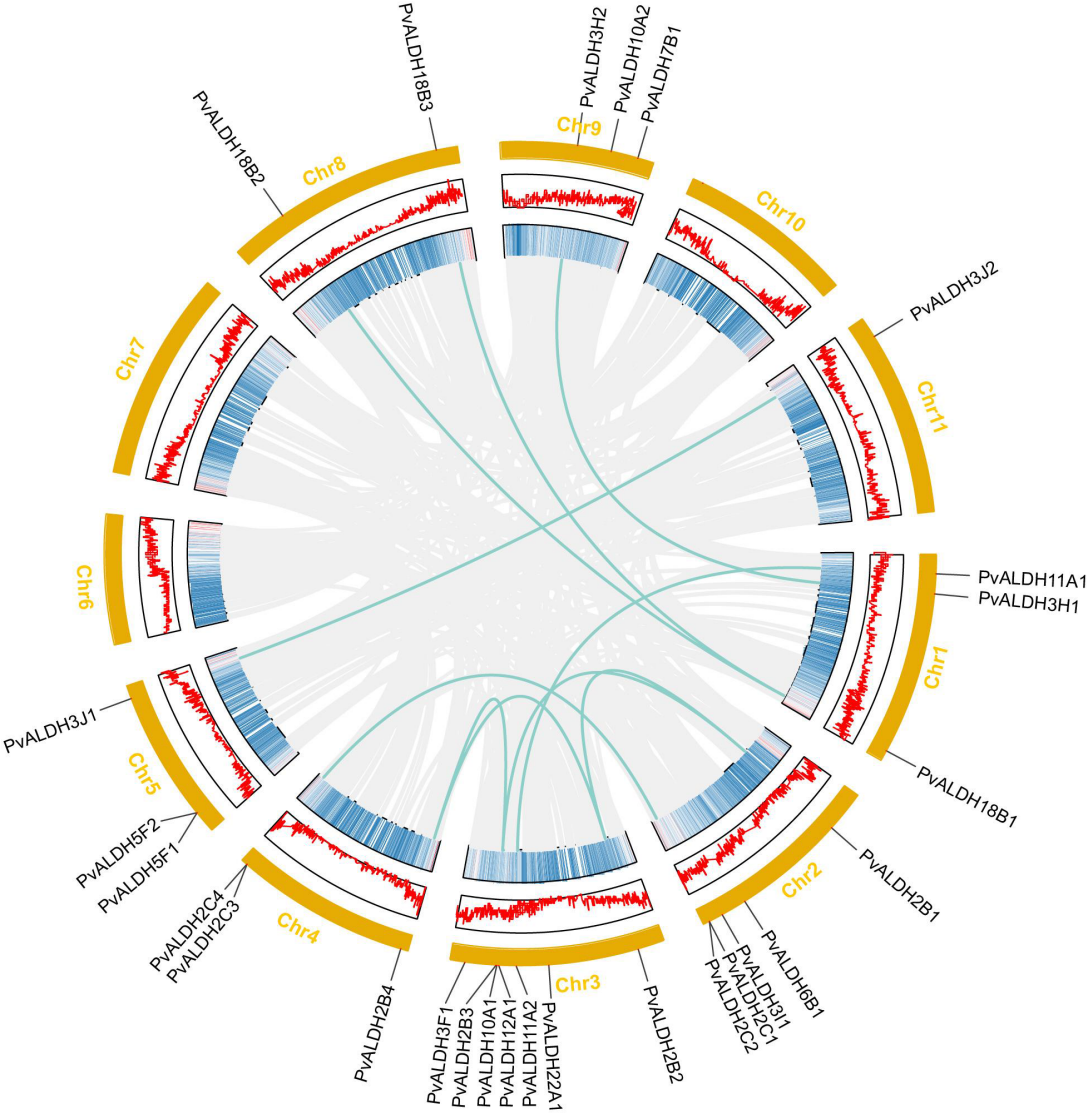
*PvALDH* gene family chromosome distribution and collinearity analysis. Common bean chromosomes are shown in orange, and *PvALDH* gene family members and their locations on the chromosome are shown in black. Each chromosome’s gene density is represented by the middle two rings. The green line represents the collinear relationship between *PvALDH* gene family members. The gray line represents the collinear background.

Nine of the eleven chromosomes in the common bean carried *PvALDHs* in an uneven distribution. A total of seven *ALDHs* were localized on chromosome 3, representing the highest count among the chromosomes. This was followed by chromosome 2 with five *PvALDHs*, chromosomes 1, 4, 5, and 9 with three each, and chromosome 8 with two. By contrast, chromosome 11 only had one *ALDH* gene and chromosomes 6, 7, and 10 had none (**Figure 5**).

In the common bean genome, 10 pairs of PvALDH genes were generated through segmental duplications: PvALDH2B1/PvALDH2B2, PvALDH2B1/PvALDH2B3, PvALDH2B2/PvALDH2B4, PvALDH2B3/PvALDH2B4, PvALDH2C1/PvALDH2C3, PvALDH3J1/PvALDH3J2, PvALDH3H1/PvALDH3H2, PvALDH11A1/PvALDH11A, PvALDH18B1/PvALDH18B2, and PvALDH18B1/PvALDH18B3. A pair of tandem duplication genes, PvALDH2C3/PvALDH2C4, were also discovered (**Figure 5**). Except for PvALDH2B1/PvALDH2B3 and PvALDH2B3/PvALDH2B4 pairs, all the duplicated PvALDH gene pairs exhibited a Ka/Ks value < 0.2. This pattern underscores the influence of purifying selection in shaping their evolutionary trajectory. The rough estimate of the time of divergence for the duplicated genes was as early as 149.35 (Mya) (PvALDH2B1/PvALDH2B3) and as late as 49.86 (Mya) (PvALDH2B1/PvALDH2B2) (**Table 1**).

**Table 1:** Gene duplication analysis of *PvALDH* genes.

No.	Locus 1	Locus 2	Ka	Ks	Ka/Ks	Duplication time (Mya)	Duplication type
1	PvALDH2B1	PvALDH2B2	0.0838	0.6082	0.1377	49.86	Segmental
2	PvALDH2B1	PvALDH2B3	2.4110	1.8221	1.3232	149.35	Segmental
3	PvALDH2B2	PvALDH2B4	0.1864	1.6242	0.1148	133.13	Segmental
4	PvALDH2B3	PvALDH2B4	2.9881	2.0763	1.4392	170.19	Segmental
5	PvALDH2C1	PvALDH2C3	0.1264	0.7194	0.1757	58.97	Segmental
6	PvALDH2C3	PvALDH2C4	0.0000	0.0000	∞	Not determinable	Tandem
7	PvALDH3J1	PvALDH3J2	0.1743	0.9791	0.1781	80.26	Segmental
8	PvALDH3H1	PvALDH3H2	0.1351	0.6850	0.1972	56.15	Segmental
10	PvALDH11A1	PvALDH11A2	0.0704	1.7200	0.0409	140.98	Segmental
11	PvALDH18B1	PvALDH18B2	0.1856	1.5302	0.1213	125.42	Segmental
12	PvALDH18B1	PvALDH18B3	0.0804	0.6303	0.1276	51.66	Segmental

Additionally, a collinearity analysis of *ALDH* genes in common bean and *Arabidopsis thaliana* was performed. The results showed that 21 collinear pairs occurred between the two species, and the most collinear members of the *PvALDH* genes were distributed on chromosome 3. These findings align with those of the collinear gene distribution in *Arabidopsis thaliana*. (**Supplementary Figure S1**).

### 3.5 Distribution and evolutionary analysis of the ALDH family

**Figure 6:**
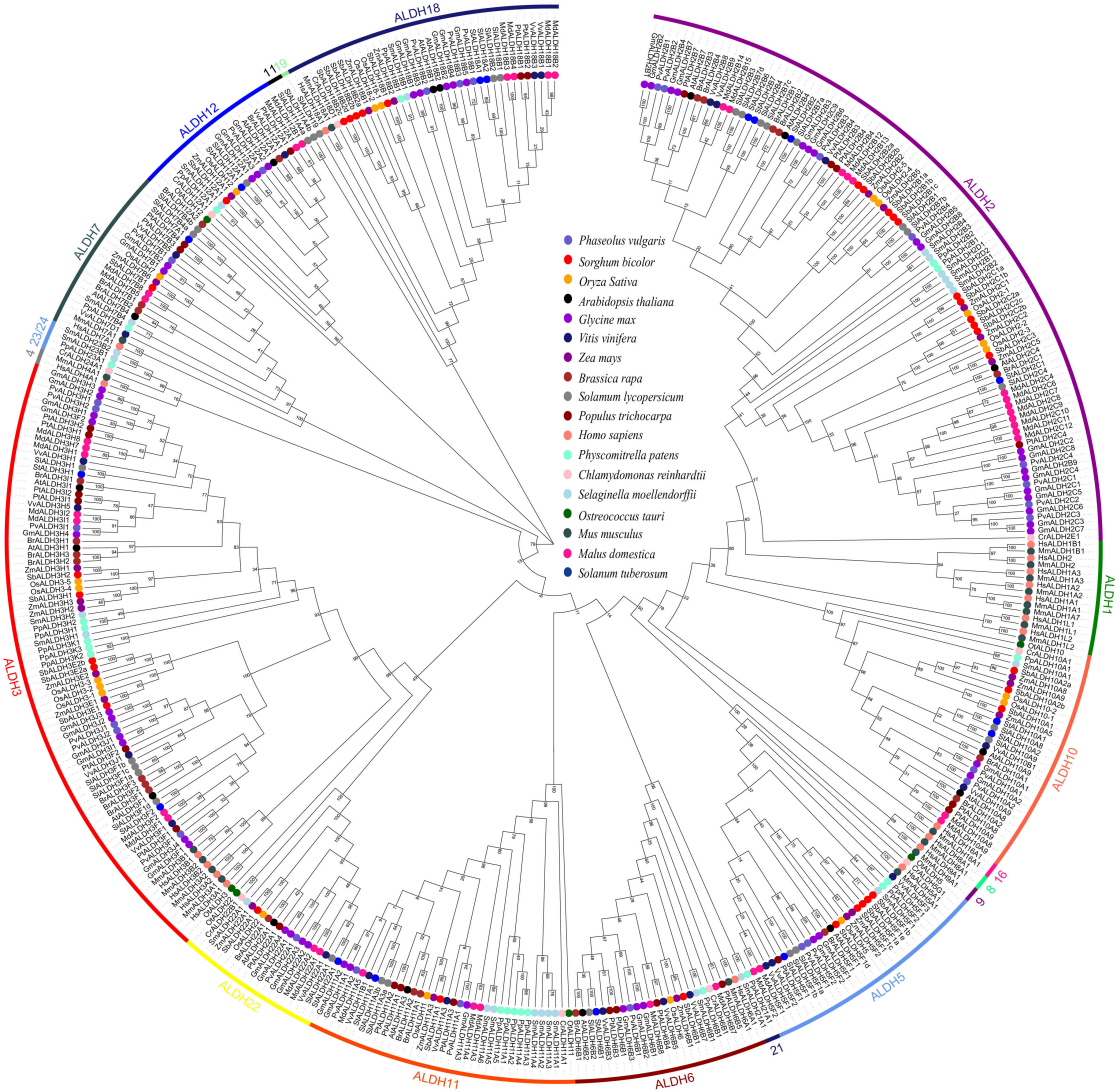
Phylogenetic analysis of the aldehyde dehydrogenase (ALDH) superfamily members. Constructing phylogenetic evolution tree using the neighbor joining (N-j) method with MEGA X. Bootstrap repeats were set to 1000, and different colored circles represent the ALDH proteins of different species in the tree.

As part of the effort to gain a better understanding of the evolutionary history of *PvALDHs*, we created a phylogenetic tree based on the protein sequences of ALDH family members from common bean and 17 other species (**Figure 6**). The 17 species include three monocots (*Sorghum bicolor*, *Oryza sativa*, and *Zea mays*), eight eudicots (*Arabidopsis thaliana*, *Malus domestic*, *Brassica rapa*, *Vitis vinifera*, *Populus trichocarpa*, *Glycine max*, *Solanum lycopersicum*, and *Solanum tuberosum*), two mosses (*Selaginella moellendorffii* and *Physcomitrella patens*), two algae (*Ostreococcus tauri* and *Chlamydomonas reinhardtii*), and two mammals (*Mus musculus* and *Homo sapiens*). The findings demonstrated that *PvALDH* genes were closely involved in eudicot species (e.g., soybean) but not in other species. All ALDH protein members from different species can be divided into 19 families (ALDH-1, 2, 3, 4, 5, 6, 7, 8, 9, 10, 11, 12, 16, 18, 19, 21, 22, 23 and 24). Most protein members from the same family exhibited clustering together, irrespective of their organismal origin or type. The phylogenetic tree also shows that the ALDH2 family has the highest number of members of all studied species, followed by ALDH3, while ALDH-5, 12, and 22 have the lowest number of members. In addition, members of the ALDH-1, 4, 8, 9, and 16 families are found only in animal species. Similarly, members of the ALDH-21, 23, and 24 families occur only in algae and mosses.

### 3.6 Motif, conserved domain, and intron–exon structure analyses of *PvALDH* proteins

**Figure 7:**
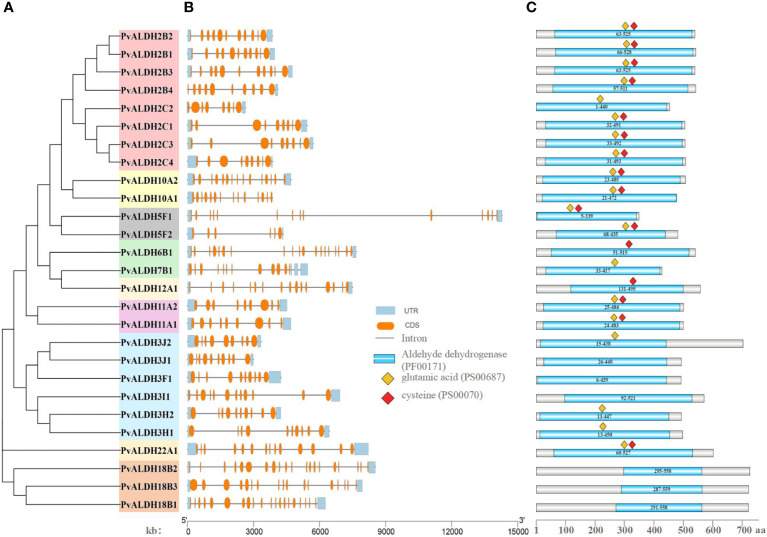
Organizational structure of common bean (aldehyde dehydrogenase) ALDH members. **(A)** Phylogenetic evolutionary tree of the PvALDH protein family. **(B)** Exon–intron distributions of the protein family. Blue represents untranslated regions (UTRs), orange represents exons, and blocking black lines represent introns. **(C)** Distribution of ALDH-conserved domains in the PvALDH proteins. The blue rectangle represents the ALDH structural domain (PF00171); The red diamond represents the active site of glutamic acid (PS00687); the yellow diamond represents the active site of cysteine (PS00070).

The protein sequences of 27 PvALDH proteins were used to construct phylogenetic trees. The data revealed that *PvALDH* proteins belonging to identical families had been clustered, which is similar to that for the phylogeny formed using ALDH members from 17 various organisms (**Figure 7A**). To clarify the structural evolution of *PvALDH* genes, the intron–exon structures of all *PvALDHs* were investigated (**Figure 7B**). We found that the number of exons in *PvALDHs* varied from 8 to 20, and genes within the same family typically had identical intron–exon structures; however, increases or loss of member exons was observed in almost all families. Among the *PvALDH* gene families, *PvALDH18B2* and *PvALDH18B1* had the highest number of exons (20), followed by *PvALDH6B1* and *PvALDH18B3* (19 and 17, respectively), while *PvALDH5F2* had the fewest exons (6). By contrast, the number of introns in *PvALDHs* varied between 5 and 19, with *PvALDH18B2* and *PvALDH18B1* containing the highest number of introns (19) and *PvALDH5F2* containing the fewest introns (5). The structural analysis of the *PvALDH* genes shows that all the genes, except *PvALDH10A1*, had upstream/downstream domains. Furthermore, with the help of Pfam, we identified the ALDH conserved domains (PF00171) of 27 PvALDH proteins (**Figure 7C**). Each PvALDH protein contained conserved ALDH domains, and the protein lengths and domain sizes of the same subfamily were similar. PvALDH22A1 had the largest ALDH domain (467 amino acids), followed by PvALDH6B1 (464 amino acids), while PvALDH18B2 had the smallest domain (263 amino acids) (**Figure 7C**).

The PvALDH protein has been analyzed for 10 conserved motifs, and they were designated as “motif1 to motif10” according to the E-values of the motifs (**Supplementary Figure S2**; **Supplementary Table S3**). The results of the analysis indicated that all PvALDH proteins contained at least two conserved motifs, as well as motif5. Therefore, motif5 is highly conserved in PvALDH family members and facilitates the maintenance of the essential functions and structures of PvALDH proteins. The motif analysis also revealed that all the members of the ALDH2 family contained 10 motifs, while the members of the ALDH18 family had the minimum number of motifs (2). Except for individual members, the motif distribution of members within a similar family exhibited similarities, indicating that PvALDH proteins in the same family may have functional similarities. In addition, among all the identified motifs, motif5 was the most common, found at 26 loci, followed by motif1 at 24 loci, whereas motif10 was the least common and found at only 8 loci (**Supplementary Table S3**).

### 3.7 Analysis of *PvALDHs* promoter CAEs

**Figure 8:**
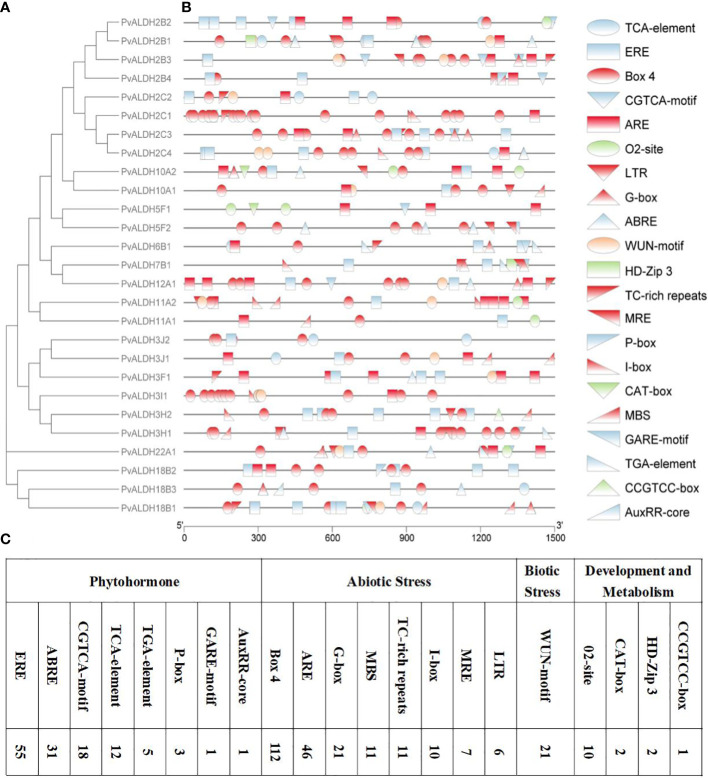
Analysis of CAEs of the *PvALDH* gene family promoters. **(A)** Phylogenetic evolutionary tree of the PvALDH protein family. **(B)** Distribution of promoter cis-acting elements in the *PvALDH* gene family. **(C)** Number of different cis-acting elements. Different colors and shapes represent different promoter cis-acting elements, and the same color represents the same type of CAEs. Blue indicates plant hormone-related response elements, red indicates abiotic stress-related response elements, green indicates development- and metabolism-related response elements, and orange indicates biological stress-related response elements.

To better understand the possible regulatory mechanisms of *PvALDHs* in the common bean under abiotic stress, PlantCARE was used to analyze the 1500-bp sequence upstream of PvALDH family members starting codons (**Figure 8**). Abiotic-responsive CAEs (n = 8), phytohormone-responsive CAEs (n = 8), development and metabolism-associated CAEs (n = 4), and biotic stress-responsive element (n = 1) were assessed in *PvALDHs* promoter regions. The results showed that all *PvALDHs*, except for *PvALDH2C1* and *PvALDH3I1*, encompassed >1 phytohormone response element (e.g., ABRE, CGTCA-motif, ERE, GARE-motif, TGA-element, P-box, AuxRR-core, or TCA-element), which indicates that different phytohormones (e.g., gibberellin, abscisic acid, auxin, salicylic acid, and jasmonic acid) have a certain impact on the expression of levels *PvALDHs* (**Figure 8A**). In addition, abiotic/biotic stress-response elements, including LTR, ARE, TC-rich element, MBS, Box 4, MRE, WUN-motif, G-box and I-box, were identified in *PvALDHs*, while abiotic stress-response elements appeared in all *PvALDHs*, with the maximum observed for Box 4 (**Figure 8B**). In general, the promoter of *PvALDH12A1* contained the highest number and types of CAEs, while the promoter of *PvALDH11A1* contained the least number and types of CAEs.

### 3.8 Expression analysis of *PvALDHs* in various developmental stages and tissues

**Figure 9:**
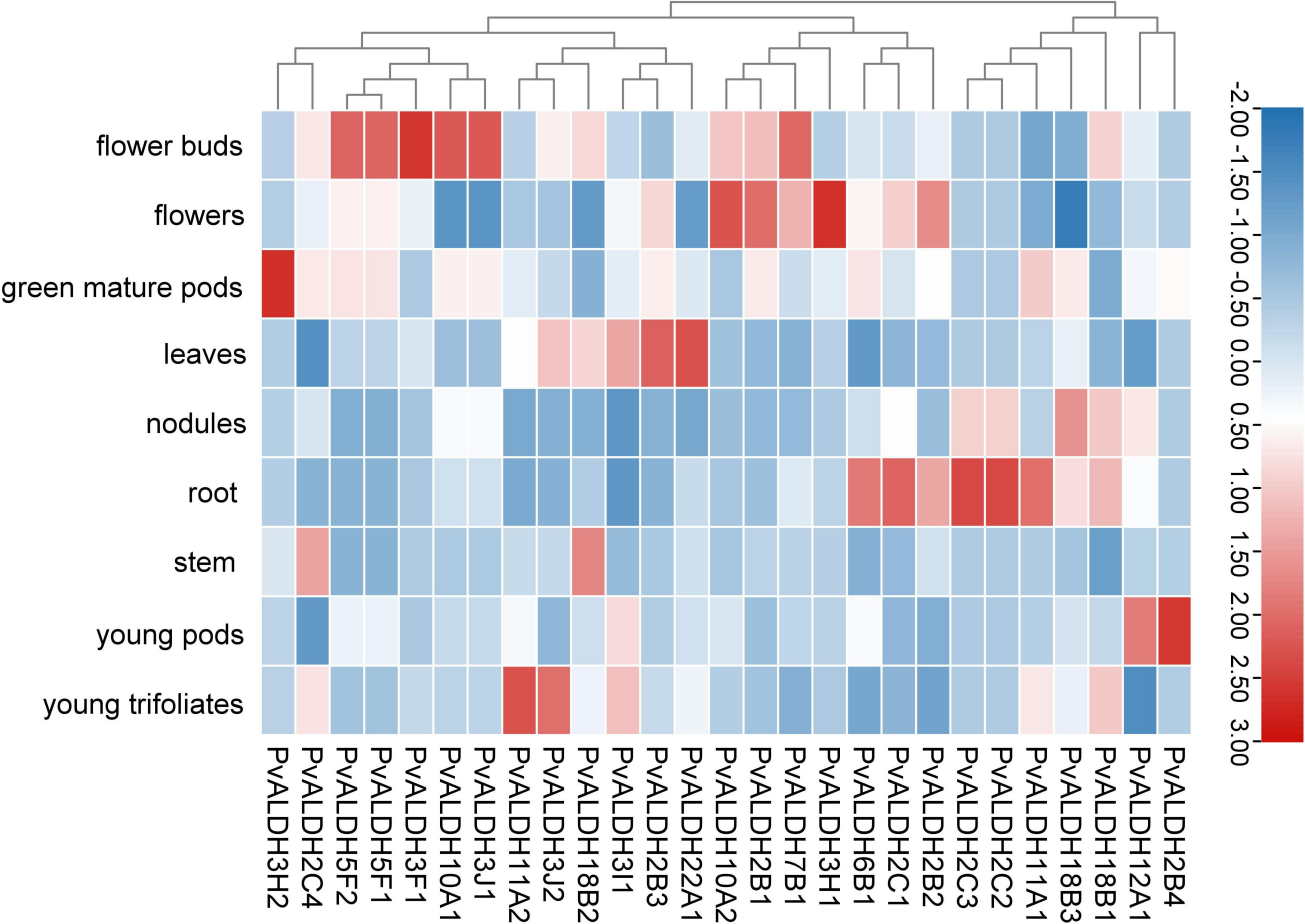
Common bean *PvALDH* gene expression in different developmental stages and tissues. Expression level is represented by different colors and intensities: deep red is the highest, deep blue is the lowest, and other colors are intermediate layers.

To clarify the role played by members of the PvALDH family in the growth and development of the common bean, the expression data of family members at various growth stages and tissues of the common bean were obtained from Phytozome, and subsequently retrieval and cluster analyses were performed (**Figure 9**; **Supplementary Table S4**). The results showed that all *PvALDHs* were expressed to varying degrees in common bean plants, with certain *PvALDHs* exhibiting tissue-specific expression. For instance, *PvALDH2B4* was upregulated only in young pods and at a low level in other tissues and organs; *PvALDH3H1* was upregulated only in the flowers and at a relatively low level in other tissues and organs; and *PvALDH22A1*, *PvALDH11A2*, and *PvALDH3H2* were upregulated only in the leaves, young trifoliates, and mature pods, respectively. Some *PvALDHs* were upregulated in various tissues. For instance, *PvALDH2C2* and *PvALDH2C3* were upregulated in the nodules and roots, whereas *PvALDH7B1*, *PvALDH10A2*, and *PvALDH2B1* were upregulated in the flowers and buds. The cluster analysis showed that numerous *PvALDH* gene family members had high expression in the buds, flowers, and roots. These findings indicate that the expression of *PvALDHs* in different tissue parts of the common bean is responsible for the gene functions; therefore, the roots, flowers, and buds may be essential tissues and organs for studying the function of *PvALDHs*.

### 3.9 Expression of *PvALDHs* under saline–alkali stress

**Figure 10:**
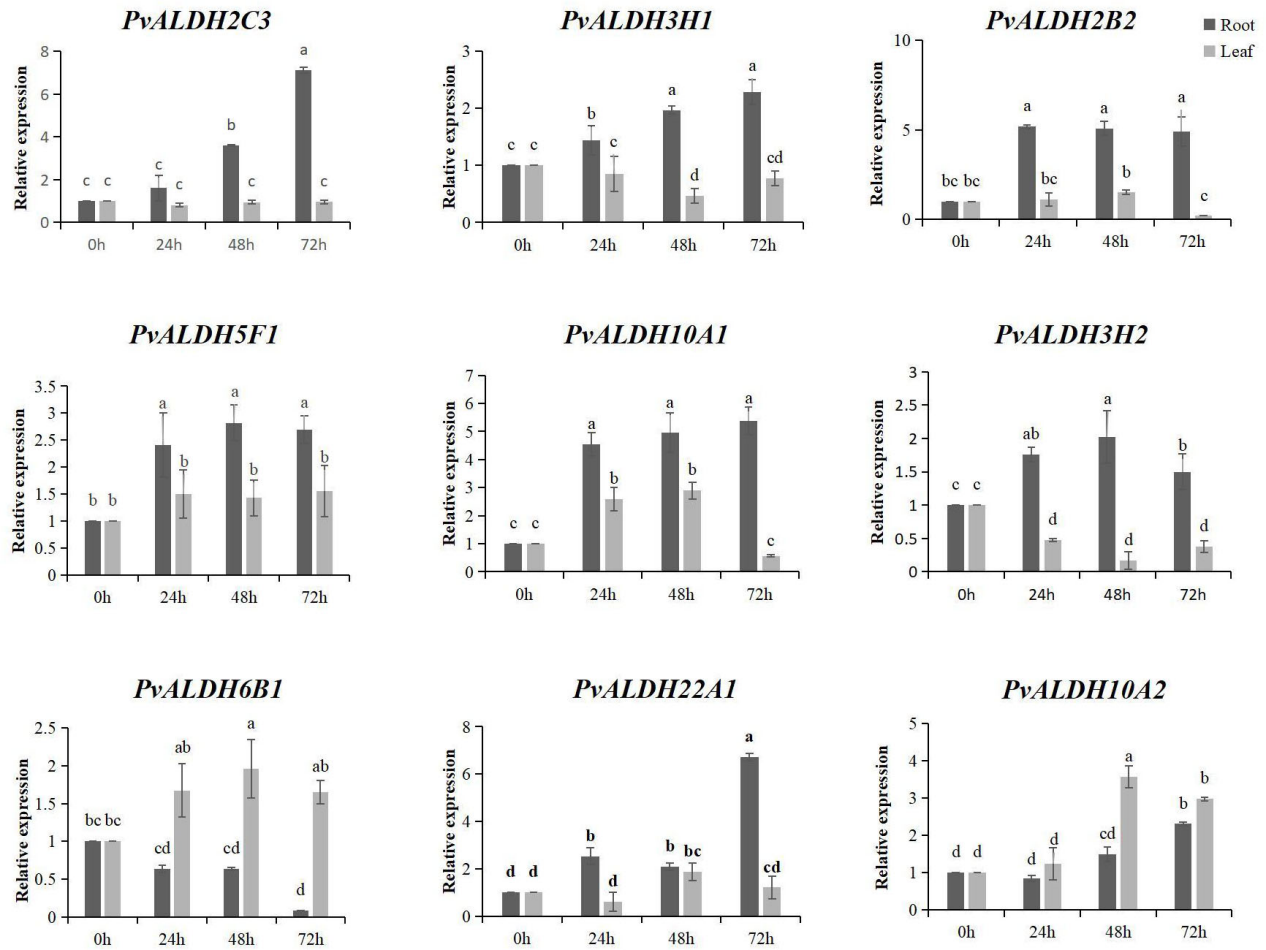
Expression pattern analysis of the *PvALDHs* under neutral salt stress. Relative expression levels of the *PvALDHs* at 24, 48, and 72 h were determined using qRT-PCR. The internal control was 0 h, and its relative expression level was 1. All data were normalized. The dark gray column indicates the expression in the root. The light gray column indicates the expression in the leaf. Different letters indicate significant differences in *PvALDH* expression levels among different tissues after stress treatment at different times (*P* < 0.05).

Nine *PvALDHs* with high expression levels were selected according to their expression patterns during different developmental stages and in different tissues. qRT-PCR was employed to detect the expression of *PvALDH* in the roots and leaves at various times under NS and AS stresses. Saline–alkali stress resulted in an imbalance in the expression levels of *PvALDHs*, and under various stress conditions, *PvALDHs* exhibited different characteristic expressions levels in the leaves and roots. Under NS stress, all other *PvALDHs*, except for *PvALDH6B1*, were upregulated in the roots, with *PvALDH2C3*, *PvALDH3H1*, *PvALDH10A1*, and *PvALDH10A2* showing a continuous upregulation pattern. However, only *PvALDH5F1*, *PvALDH6B1*, *PvALDH10A2*, and *PvALDH22A1* were upregulated in the leaves after exposure to neutral salt stress for 72 h (**Figure 10**; **Supplementary Table S5**).

**Figure 11:**
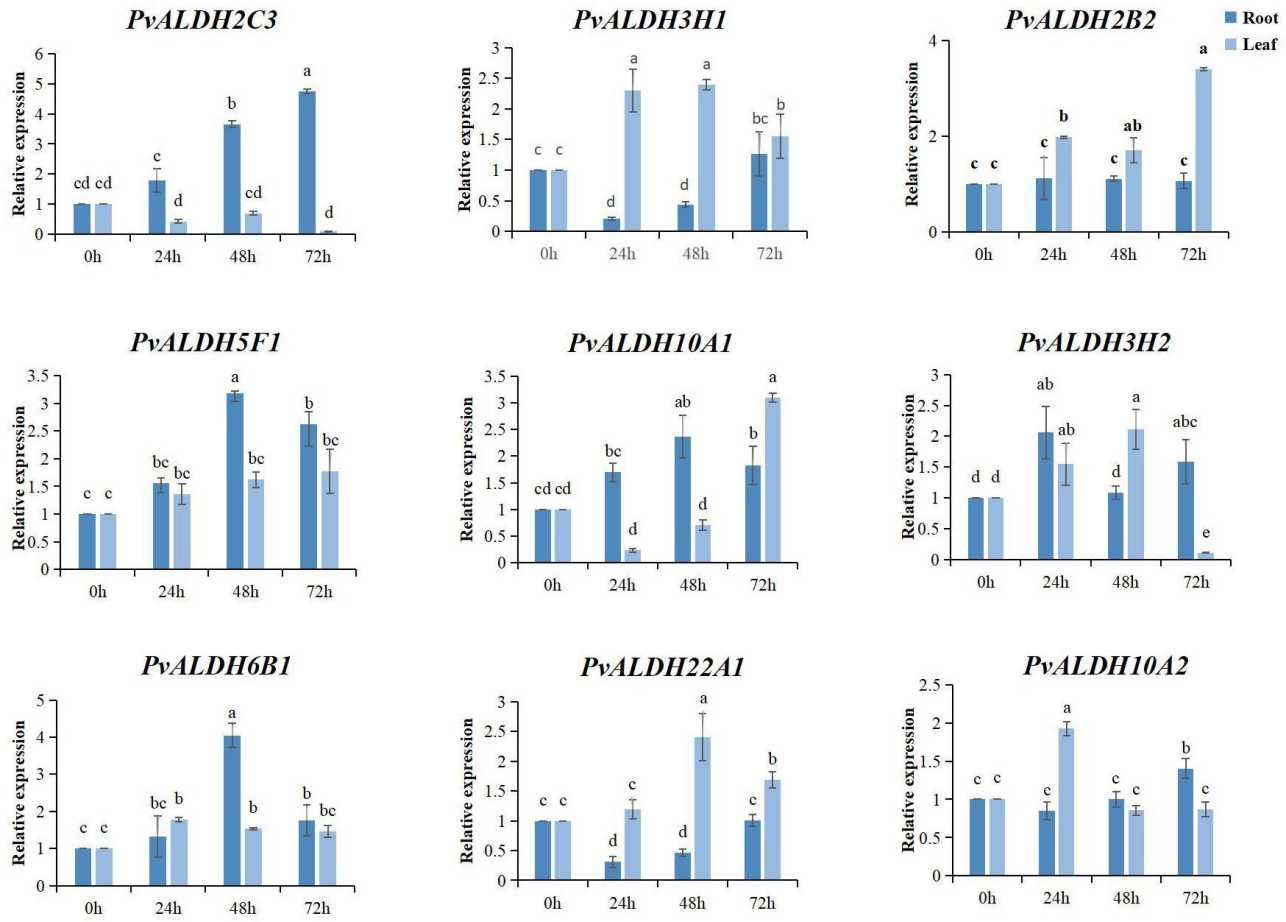
Expression pattern analysis of the *PvALDHs* under alkaline salt stress. Relative expression levels of the *PvALDHs* at 24, 48, and 72 h were determined using quantitative real-time polymerase chain reaction. The internal control was 0 h, and its relative expression level was 1. All data were normalized. The dark blue column indicates the expression in the root. The light blue column indicates the expression in the leaf. Different letters indicate significant differences in *PvALDH* expression levels among different tissues after stress treatment at different times (*P* < 0.05).

After 72 h of AS stress, all nine *PvALDHs* were upregulated in the roots; however, only *PvALDH2C3* continued to be upregulated at all treatment times. Moreover, *PvALDH10A1*, *PvALDH5F1*, and *PvALDH6B1* were upregulated after 48 h of AS stress. *PvALDH3H1*, *PvALDH2B2*, *PvALDH10A1*, and *PvALDH22A1* were significantly upregulated in the leaves after 72 h of stress, whereas *PvALDH2C3* and *PvALDH3H2* were significantly downregulated (**Figure 11**; **Supplementary Table S5**). These results indicate that *PvALDH* gene family members have different responses and regulatory mechanisms under saline–alkali stress.

### 3.10 Homology modeling of different PvALDH proteins

**Figure 12:**
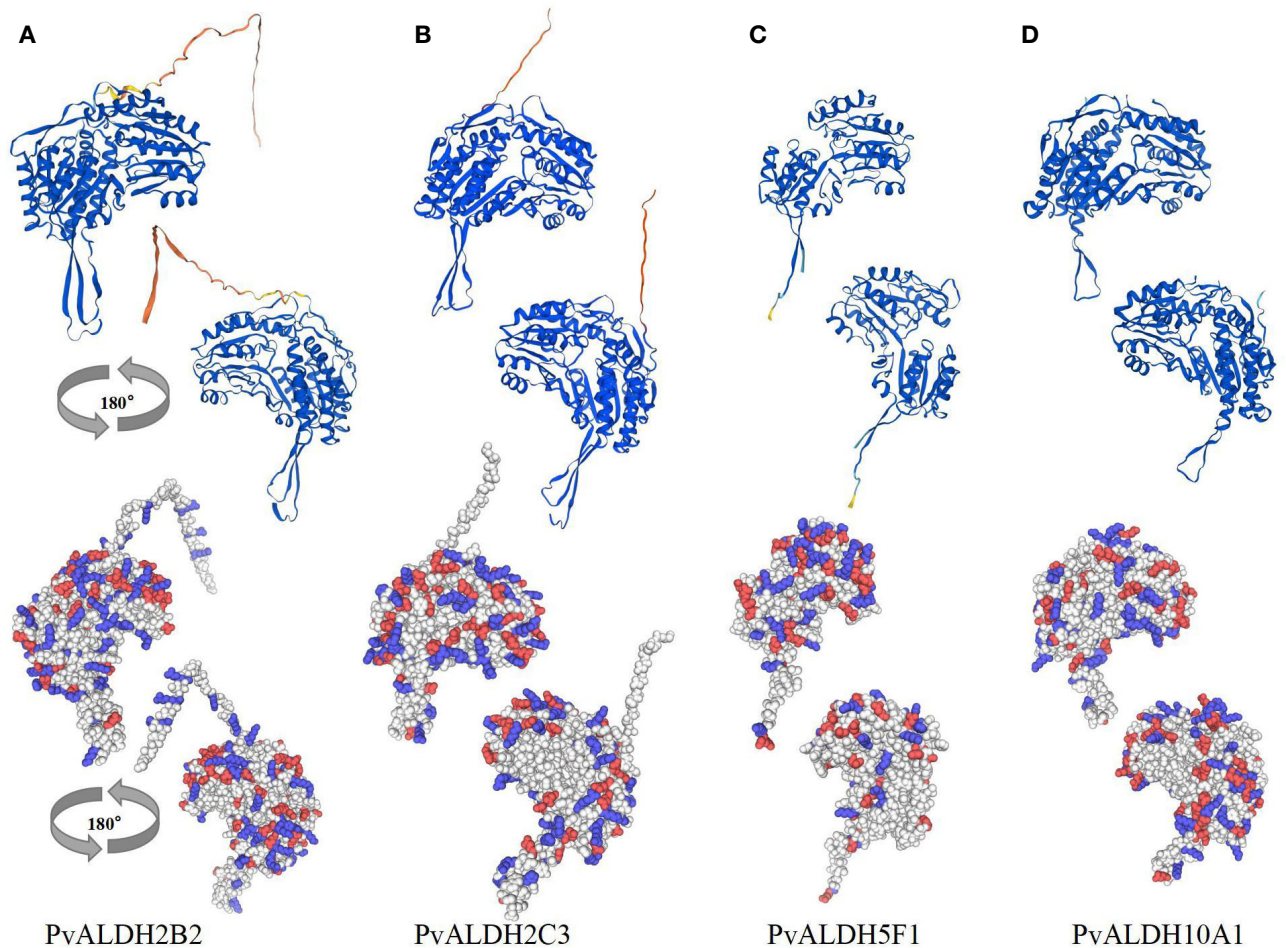
PvALDH protein three-dimensional structure analysis. Four neutral salt and alkaline salt stress-specific proteins, **(A)** PvALDH2B2, **(B)** PvALDH2C3, **(C)** PvALDH5F1, and **(D)** PvALDH10A1 were selected for homology modeling. By analyzing each protein’s structure, cartoon diagrams were created in two different views (rotated 180°). The surface-charge distribution of the selected proteins was identified, with red and blue denoting negatively and positively charged amino acids, respectively.

The Self-Optimized Prediction Method with Alignment was employed to forecast varying ratios of extended strands, alpha helices, coils, and beta turns within distinct PvALDH protein structures (**Supplementary Table S6**). Among the protein secondary structure predictions, the spiral structure accounted for the largest proportion (36.69–49.16%), followed by the random coil (26.99–41.05%), extended strand (13.40–19.13%), and beta-turn structures (4.70–8.92%). Protein glycosylation plays an important role in modifying protein structure and can affect various biological processes, including protein folding, signal transduction, transportation, cell–cell interaction, and immune response (Rudd et al., 1999; Brennan et al., 2011). Glycation analysis and prediction were conducted on the 27 PvALDH proteins, and 15 PvALDH proteins were found to have potential N-glycosylation sites. PvALDH3F1, PvALDH18B1, and PvALDH22A1 were dominant in terms of quantity and exhibited four N-glycosylation sites (**Supplementary Table S7**). To further understand the structural organization and three-dimensional coordination of ALDH in the common bean, the four saline–alkali stress-responsive proteins PvALDH2B2, PvALDH2C3, PvALDH5F1, and PvALDH10A1 were chosen for homology modeling using the templates of cowpea (*Vigna unguiculata*; AFDB: A0A0S3R2B5.1.A), peanut (*Arachis hypogaea*; AFDB: A0A445D2Y8.1.A), common bean (*Phaseolus vulgaris*; AFDB: V7BT57.1.A) and narrow-leaved lupine (*Lupinus angustifolius*; AFDB: A0A4P1R3F2.1.A) ALDHs, respectively (**Figure 12**). MolProbity Ramachandran was then used to plot and verify the accuracy of the generated homologous model (**Figure S3**). The results revealed that the residues of PvALDH2B2 (**Figure 12A**), PvALDH2C3 (**Figure 12B**), PvALDH5F1 (**Figure 12C**), and PvALDH10A1 (**Figure 12D**) were placed in favorable regions at 94.4, 97.4, 97.4, and 97.0%, respectively. This result also confirms the accuracy and effectiveness of homology modeling. The diagram generated from homology modeling demonstrated a high degree of structural similarity among the four chosen proteins, particularly regarding shared strands and helices within the Rossmann folding motif (**Figure 12**). In addition, the analysis of the charge distribution on the selected protein surface showed significant differences in the distribution of positively and negatively charged amino acids on different protein surfaces (**Figures 12A–D**).

## 4 Discussion

Soil salinization is a fundamental abiotic stressor that hinders plant growth, posing a substantial obstacle to the sustainable advancement of the environment and agriculture (Martínez-Cuenca et al., 2013; Guo H. et al., 2020). Salt and alkali stresses can cause adverse effects, such as metabolic disorders, root development inhibition, and photosynthesis performance decline in plants, remarkably decreasing crop yield (Nam et al., 2012; Mo et al., 2020). Approximately 95% of plant biomass accumulation comes from photosynthesis, and inhibition of photosynthesis under stress is one of the key mechanisms that hinder plant growth (Wang et al., 2021). Inhibition of photosynthesis leads to an excess of electrons and energy in the photosynthetic electron transport chain, resulting in a ROS burst and peroxidative damage (Zhang et al., 2020). In this study, compared to those in the control group, the *Tr*, *Ci*, *Gs*, and *Pn* of common bean leaves were significantly reduced NS–AS stresses (**Figures 1D–H**). This result is consistent with the response of photosynthesis in *Medicago ruthenica* seedlings to NS–AS stress (Li et al., 2010). NS–AS stress induces the production of ROS, such as H_2_O_2_, O_2_^•-^, and •OH in plants, while SOD, CAT, and POD are key protective enzymes to maintain the balance of reactive oxygen species metabolism in the cell, as they can decompose and convert reactive oxygen species into harmless substances (Li et al., 2008). The accumulation of ROS in plant cells can produce oxidative effects on macromolecules, such as cell membranes, nucleic acids, and biological enzymes, disrupting normal physiological processes (Zhao et al., 2019). Under this experimental condition, and compared with those observed in the control treatment group, the levels of O_2_^•-^, H_2_O_2_, and MDA in the roots and leaves were markedly increased under NS–AS stress (**Figures 2A, E, F**), indicating that the NS or AS stress treatment caused damage to the cell membranes of common bean plants (Apel and Heribert, 2004). In addition, under NS–AS stress, we observed an increase in the levels of SOD and POD in the leaves; similarly, the levels of SOD, POD, and CAT in the roots were significantly increased. Compared with those under NS stress, the increase in SOD and POD activities under AS stress was more significant (**Figures 2B–D**). This finding indicates that compared to NS stress, severe oxidative damage cannot be avoided due to the large accumulation of ROS, although AS stress induces higher levels of antioxidant enzyme activity in the common bean. In addition, our findings indicate that AS stress leads to a marked decline in photosynthesis and diminished dry matter accumulation, consequently exerting a more profound inhibitory effect on plant growth, compared with NS stress.

In response to environmental stress hazards, plants produce numerous ROS and toxic compounds, resulting in the accumulation of intracellular aldehydes (Shin et al., 2009; Singh et al., 2013). Lipid peroxidation and respiration induce in the formation of reactive aldehydes, such as MDA and acetaldehyde (Shin et al., 2009). Reactive aldehydes readily form adducts with DNA, RNA, and proteins, leading to impaired cellular homeostasis, enzyme inactivation, DNA damage, and cell death (Nadkarni and Sayre, 1995; Brooks and Theruvathu, 2005; Jacobs and Marnett, 2010). ALDH enzymes use either NAD+ or NADP+ as a cofactor to convert aldehydes to their corresponding carboxylic acids plus NADH or NADPH. Moreover, the ALDH-mediated generation of NADH/NADPH represents a major source of reducing equivalents required for maintaining cellular redox balance (Brocker et al., 2013). In the presence of salt–alkali stress, plants initiate a process where the root system detects the stress signals and progressively conveys them to the above-ground tissues (Fang et al., 2021). The analysis of the change in the trend of ALDH enzyme activity in the common bean root under NS or AS salt stresses showed that with increased stress treatment time, ALDH enzyme activity markedly increased significantly (**Figure 3**). Moreover, it continued to increase under neutral salt stress. These findings indicate that the ALDH enzyme actively participates in the response and adaptation process of common bean roots to NS and AS stresses. Therefore, this observation further indicates that the ALDH enzyme can effectively transform excess aldehyde in organisms and maintain stable intracellular metabolism. In addition, we found that compared to AS stress, NS stress induced higher ALDHase activity to promote a better response of common beans to the adverse environment. This finding suggests that under AS stress, ALDH enzyme activity may actively participate in the catabolism of toxic aldehydes produced by oxidative damage to the cell membrane induced by ROS accumulation in common bean, reducing the inhibition of enzyme activity and DNA damage by reactive aldehydes and thus alleviating the degree of impaired cellular homeostasis. Therefore, the root may be an important target for studying the functions of the *PvALDHs*.

Different studies have that identified ALDH family members in a variety of species (Brocker et al., 2013), such as 16 *AtALDH* members in Arabidopsis thaliana (Hou and Bartels, 2015), 53 *AtALDHs* members in soybean (Wang et al., 2017), 22 members in maize (Jimenez-Lopez et al., 2010), 20 members in rice (Gao and Han, 2009), 39 members in apples (Li et al., 2013), 29 members in tomatoes (Jimenez-Lopez et al., 2016), 23 members in grapes (Zhang et al., 2012), 19 members in sorghum (Islam et al., 2022), and 22 members in potatoes (Islam et al., 2021). Herein, we discovered 27 PvALDH family members from the common bean (**Supplementary Table S2**). Compared with that for Arabidopsis (16) and rice (20), the common bean has higher numbers of ALDH family members, although compared with soybean (53), the number is lower, which may be related to the size and evolution of the genomes of the different species (Zhang et al., 2023). Gene duplication constitutes a significant phase in the enlargement of gene clusters, with tandem and segmental duplications emerging as the central mechanisms for this process (Cannon et al., 2004). The position and sequence similarity of *PvALDHs* on chromosomes were analyzed, and both tandem and fragment duplications were responsible for the expansion of the *PvALDH* genes, with fragment duplication playing a considerable role (**Figure 5**; **Table 1**). These results align with previous reports on rice (Gao and Han, 2009), apples (Li et al., 2013), and grapes (Zhang et al., 2012). In addition, *PvALDH2B1*, *PvALDH2B2*, *PvALDH2B3*, and *PvALDH2B4* were collinear with *AtALDH2B7* (AT1G23800.1), a gene in Arabidopsis that responds to abscisic acid and drought signals (Depuydt and Vandepoele, 2021). *PvALDH10A2* is collinear with *AtALDH10A8* (AT1G74920), a gene in Arabidopsis that responds to drought and salt stresses (Missihoun et al., 2011). This finging suggests that *PvALDHs* have similar functions in common beans and *Arabidopsis Thaliana* and *PvALDHs* are crucial in coping with some abiotic stresses in common beans.

An evolutionary relationship analysis was performed to classify the ALDH family members of the common bean (**Figure 6**), and the PvALDH family members were classified as 10 families (ALDH-2, 3, 5, 6, 7, 10, 11, 12, 18, and 22). Although 14 ALDH families have been discovered in plants, only 10 ALDH families exist in the common bean and other angiosperms, indicating that these 10 families evolved before the differentiation of monocotyledonous and dicotyledonous plants. By contrast, ALDH-19, 21, 23, and 24 were only found in certain plants, and members in the ALDH19 family were only present in tomatoes, indicating that this family played a crucial role in the evolutionary process of this plant (Jimenez-Lopez et al., 2016). Members of the ALDH21 and ALDH23 families were only found in Selaginella moellendorfii and Physcomitrella patens (Chen et al., 2002), while ALDH24 was only found in Chlamydomonas reinhardtii (Wood and Duff, 2009). These results indicate that the ALDH-21, 23, and 24 families may have played a crucial role in the shift from aquatic to terrestrial plants but appear to have become lost during angiosperms evolution. Additional *ALDH* gene groups were observed in the phylogenetic tree, such as ALDH-1, 4, 8, 9, and 16, which have only been found in mammalian species (such as humans and mice) and have not been found in any plant species.

It may be possible to link changes in exon–intron numbers with gene fragment fusions and rearrangements, indicating that changes to gene structure play a central role in gene family evolution (Xu et al., 2012). This research determined that the structure of *PvALDHs* in different families showed considerable differences, although the number of exons in *PvALDHs* within the same subfamily was comparable (**Figure 7**), indicating that *PvALDHs* had certain differences among different families. The potential functions of genes can be revealed using a motif analysis of protein sequences because motifs of similar types may exert comparable functions (Bailey et al., 2015; Liu et al., 2021). The motif analyses of PvALDH family members indicated that members of the same subfamily had comparable motifs (**Supplementary Figure S2**), and these findings are consistent with those reported on soybean (Wang et al., 2017).

Previous reports have suggested that CAEs elements in plant promoters are core factors for transcriptional regulation and play an essential role in modulating the molecular networks of various biological processes, such as abiotic stress responses, phytohormonal responses, and development (Yamaguchi-Shinozaki and Shinozaki, 2005; Ibraheem et al., 2010). The analysis of CAEs in common bean promoters showed that they are related to phytohormones (ERE, ABRE, P-box, GARE-motif, CGTCA-motif, AuxRR-core, TCA-element, TGA-element) and abiotic stress (LTR, ARE, TC-rich element, MBS, Box 4, MRE, I-box, G-box) in *PvALDHs*, suggesting that the function of *PvALDHs* may be associated with abiotic stress and phytohormonal responses (**Figure 8**). Similarly, CAEs such as MBS, LTR, TCA-element P-BOX, and ABRE, have also been found in *SbALDHs*, *StPvALDHs*, and *GhALDHs* (Guo et al., 2017; Islam et al., 2021, 2022). Therefore, MBS, LTR, TCA-element, ARBE, and ARE are common CAEs in ALDH family members and they contribute to certain functions of PvALDHs in the interaction between abiotic stress and phytohormone responses (Zhu et al., 2014).

The expression analysis of PvALDH family members in various developmental stages and tissue parts of the common bean revealed that *PvALDHs* exhibited high expression levels in the roots (**Figure 9**), which is similar to the results of the tissue-specific expression analyses of *GhALDHs*, *GmALDHs*, and *SiALDHs* (Dong et al., 2017; Wang et al., 2017). The function of *ALDHs* has been thoroughly examined in various plants, and the family members are involved in several biosynthetic and catabolic pathways. For example, members of the ALDH2 family are involved in the metabolism of acetaldehyde, whereas ALDH6 family constituents act as methyl-malonyl-semialdehyde-dehydrogenases, facilitating reactions associated with pyrimidine and valine catabolism (Brocker et al., 2013). Apart from their roles in different metabolic and biosynthetic aspects, several *ALDHs* are responsible for coping with diverse abiotic stresses, including salinity, drought, cold, heat, ABA, and PEG (Huang et al., 2008; Stiti et al., 2011; Chen et al., 2015). According to previous reports, *ALDH* genes across a wide range of plant or crop species displayed similar fluctuations in expression patterns under varying abiotic stresses. For example, under drought stress, *OsALDH2–4* in rice and *GmALDH2B2* in soybean are both upregulated (Gao and Han, 2009; Wang et al., 2017). Under salinity, drought, and heat stress, *StALDH12A1*, *StALDH7A1*, and *StALDH2B6* transcripts (BARI Alu-7) were upregulated in a Bangladeshi potato variety (Islam et al., 2021). In this study, *PvALDH2C3*, *PvALDH5F1*, and *PvALDH10A1* were significantly upregulated in the roots under NS or AS stresses, while only *PvALDH6B1* was significantly downregulated under neutral salt stress (**Figures 10**, **11**). Combined with the prediction of N-glycosylation sites of the PvALDH protein, the aforementioned *PvALDHs* play crucial roles in the response of the common bean to neutral or alkaline salts or other abiotic stresses.

## 5 Conclusions

Compared with NS stress, AS stress induces higher levels of antioxidant enzyme activity in the common beans, however, severe oxidative damage remains inevitable due to the substantial accumulation of ROS. Moreover, photosynthetic performance is markedly impaired, with reduced dry matter accumulation, which substantially inhibits plant growth. The ALDH enzyme in common beans actively responds to NS or AS stresses by inducing the expression of *PvALDH* genes. Furthermore, a total of 27 ALDH family members were discovered from the common bean reference genome and grouped into 10 families. *PvALDHs* within a similar subfamily had comparable motifs and gene structures. *ALDHs* in common bean have expanded based on segmental and tandem duplications, with segmental duplication playing a dominant role. A collinearity analysis indicated that *PvALDHs* may be necessary for abiotic stress response, and abiotic stress and phytohormone response CAEs are also found in promoter regions. *PvALDHs* exhibit tissue-specific expression in the different developmental stages and parts of common beans, and the root system is an important target tissue for further studies on *PvALDHs*. Additionally, the levels of *PvALDH2C3*, *PvALDH5F1*, and *PvALDH10A1* in common bean root were markedly upregulated, while PvALDH6B1 was markedly downregulated under NS–AS stress, which further indicated that *PvALDHs* play a regulatory role in plant response to NS–AS stress. This study adds to the theoretical knowledge of *ALDHs* in common bean and provides a foundation for further investigations of the function of common bean *ALDHs* in abiotic stress responses.

## Data availability statement

The datasets presented in this study can be found in online repositories. The names of the repository/repositories and accession number(s) can be found in the article/**Supplementary Material**.

## Author contributions

XW: Writing – original draft, Data curation, Formal Analysis, Investigation, Methodology, Software, Visualization. MW: Data curation, Formal Analysis, Investigation, Methodology, Software, Writing – original draft, Project administration, Resources. SY: Project administration, Writing – original draft, Conceptualization, Funding acquisition, Supervision, Writing – review & editing. LZ: Data curation, Formal Analysis, Investigation, Methodology, Resources, Software, Writing – original draft. XZ: Data curation, Investigation, Methodology, Resources, Writing – original draft. LY: Conceptualization, Funding acquisition, Project administration, Supervision, Writing – review & editing. YZ: Conceptualization, Funding acquisition, Project administration, Supervision, Validation, Writing – original draft.
