# Supplementary material for: Comprehensive analysis of the aldehyde dehydrogenase gene family in Phaseolus vulgaris L. and their response to saline–alkali stress
Source: Front Plant Sci. 2024 Feb 21;15:1283845. doi: 10.3389/fpls.2024.1283845 (PMC10915231; doi:10.3389/fpls.2024.1283845)
Supplement: Supplementary file 1 [file DataSheet_1.docx]

Supplementary Material

Comprehensive Analysis of the Aldehyde Dehydrogenase Gene Family in *Phaseolus vulgaris* and Their Response to Saline–Alkali Stress

Xiaoqin Wang^1^, Mingxu Wu^1^, Song Yu^1, 3*^, Lingxia Zhai^1, 2,^ Xuetian Zhu^1^, Lihe Yu^1, 3^, Yifei Zhang^1, 3^

*** Correspondence:** Song Yu: yusong@byau.edu.cn

# Supplementary Figures and Tables

## Supplementary Figures


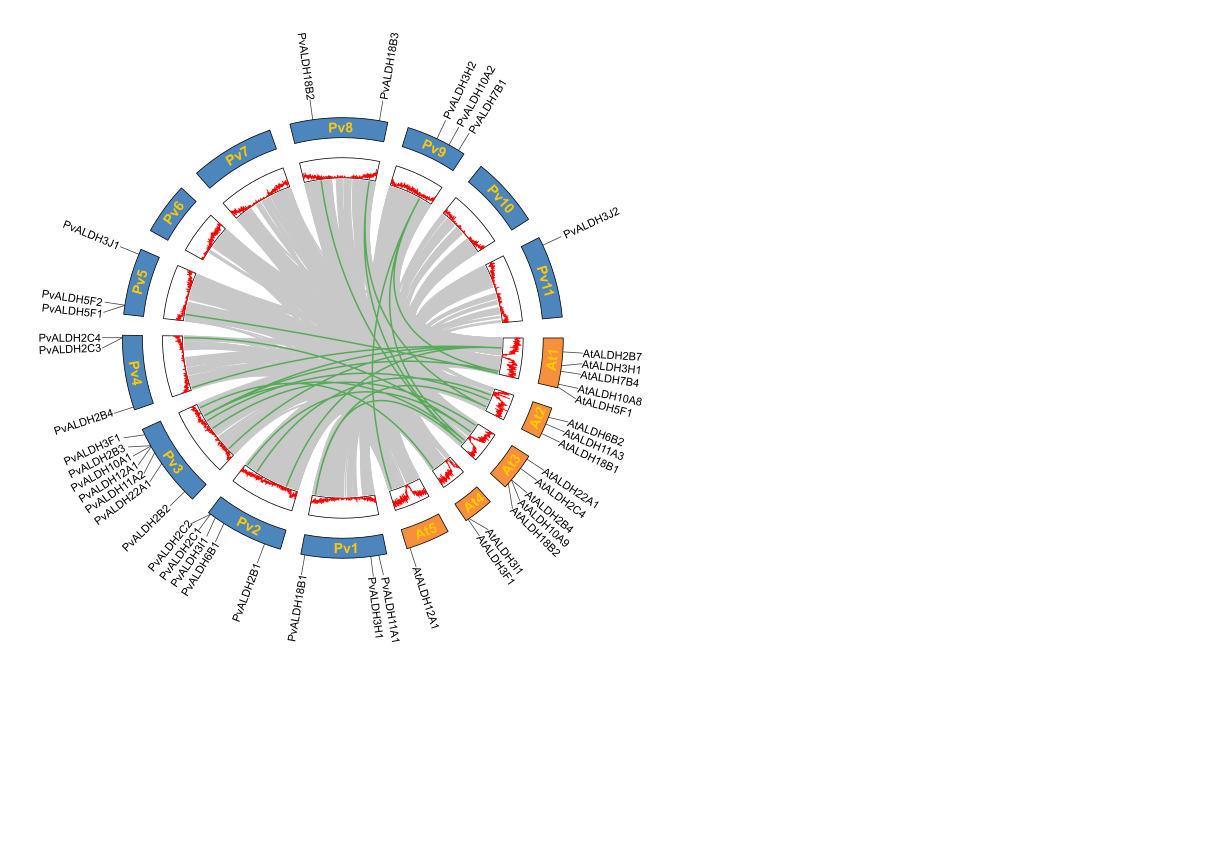


**Figure S1.** Collinear analysis of *ALDH* genes in common bean and *Arabidopsis thaliana*. The green line shows the collinearity of *ALDH* genes in common bean and *Arabidopsis thaliana.* The gray line shows the collinear backgrounds between the two species, and the red line shows the chromosome density.


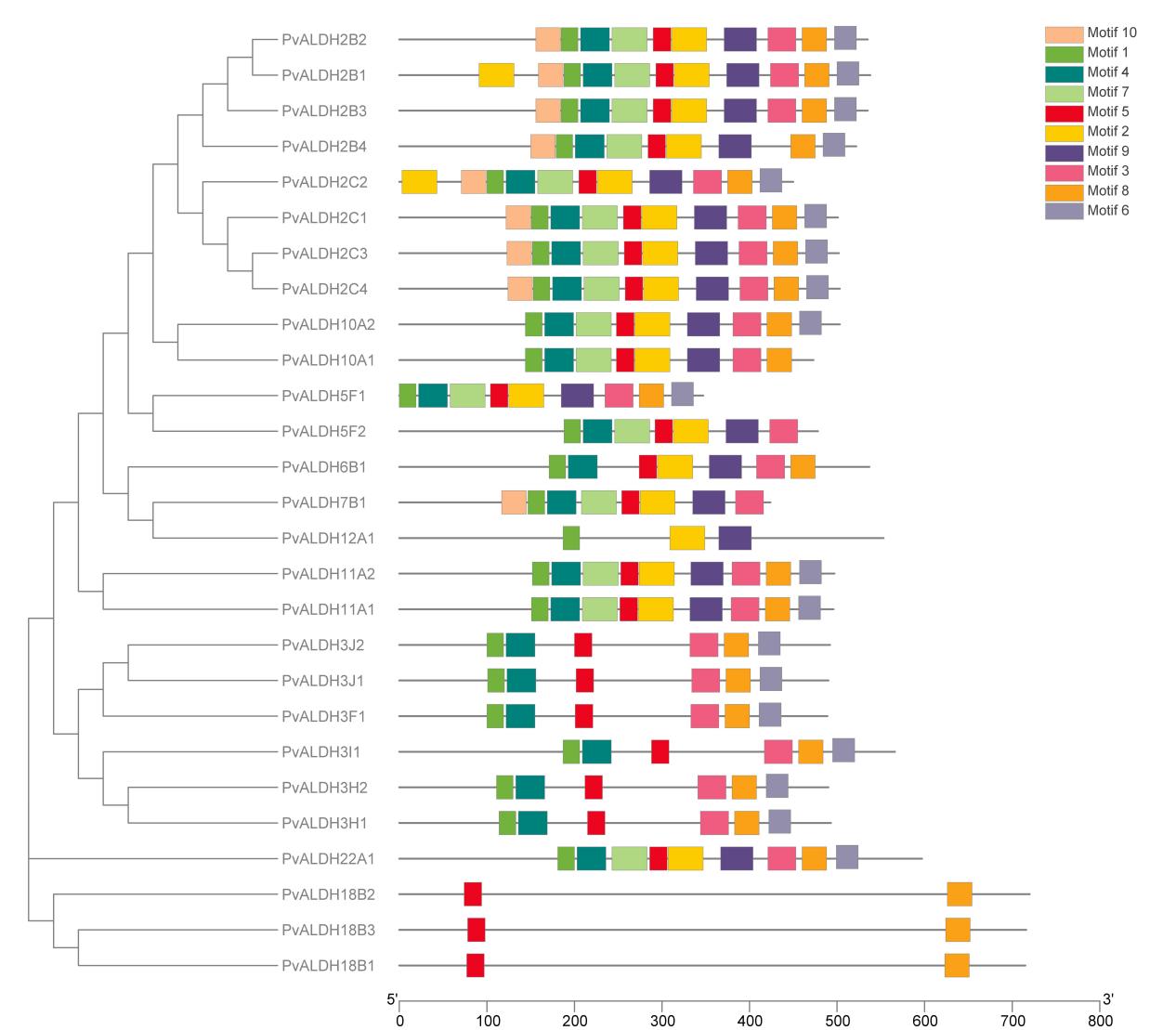


**Fig****ure S2.** Distribution of conserved motifs in the PvALDH protein family. Different colored boxes indicate different conserved motifs


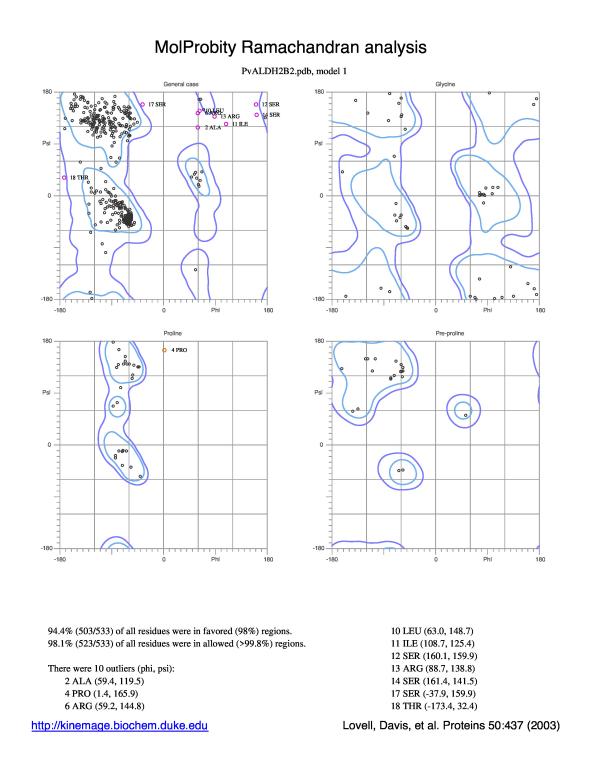

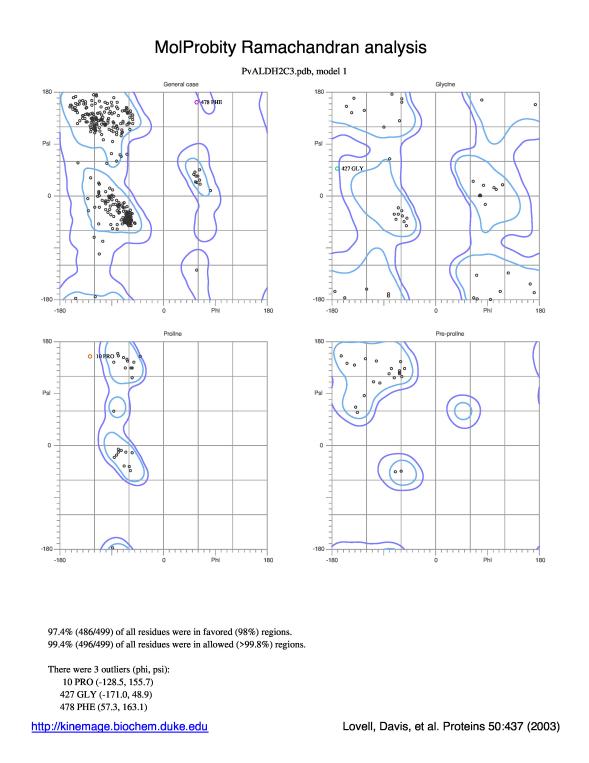


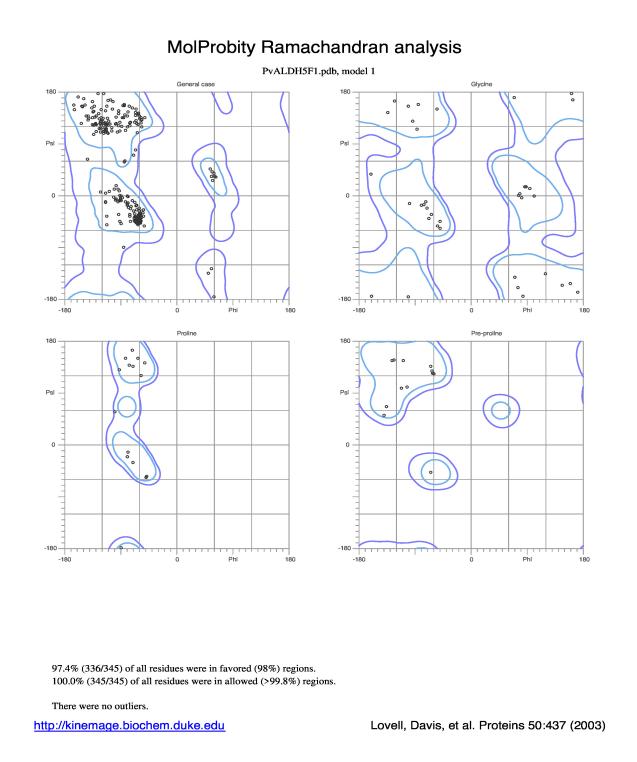

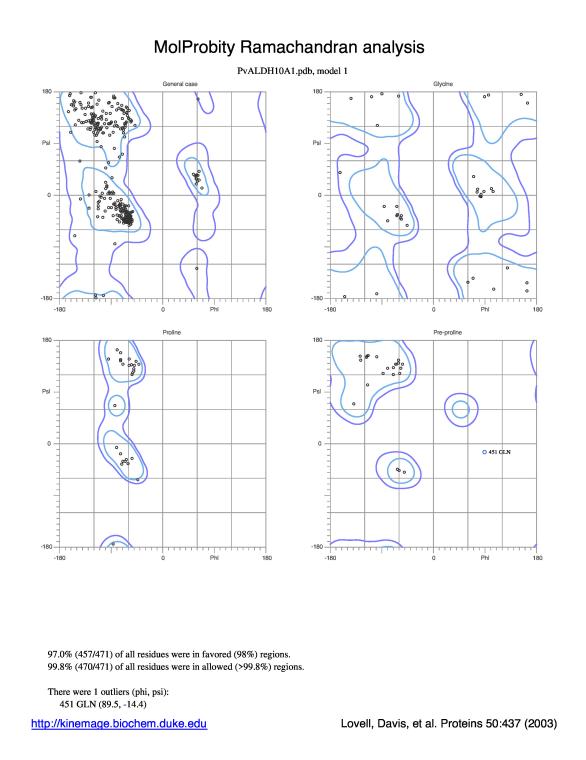


**Figure S3.** MolProbity Ramachandran analysis. Ramachandran plot analysis to validate the 3D homology model of the four selected PvALDH proteins. Favorable and allowed regions of different cases are shown in the figure and were verified based on a MolProbity Ramachandran analysis using PSVS (https://montelionelab.chem.rpi.edu/)

## Supplementary Tables

**Table S1.** Primers used for the real-time quantitative reverse transcription polymerase chain reaction analysis

| **Primer Name** | **Forward Primer (5′→3′)** | **Reverse Primer (5′→3′)** |
| --- | --- | --- |
| *PvALDH2B2* | AAGGCTCGTGCTTTGAAACG | TTGTCGCCAAGTCTGTCTCC |
| *PvALDH2C3* | CTGCAGGTTGCACAATGGTC | TTGCAGCACCTGCAGTTAGA |
| *PvALDH3H1* | GTTGGCAAAACTGCTGGGAG | GCAGCAGCCATCACAATACG |
| *PvALDH3H2* | CGGGAAGGACGCGAAGTTAT | ACGAGGGTTTCGAGTGAAGG |
| *PvALDH5F1* | ATGTGGCTGTACAGTGGTCG | TGCAAGTAAAGCGTCCCCAA |
| *PvALDH6B1* | TTAGAGGTGGTGGAACACGC | ACAGGGAACATCCACAAGGG |
| *PvALDH10A1* | GCGGTTTTGGTCGAGAACTAGG | CGGTTCATCAGAGATGTACTGAGTC |
| *PvALDH10A2* | AGCAAAAGGCCCCTGTATCC | CAGTACAACCAGCAGCCAGA |
| *PvALDH22A1* | ATCCAATCAGCCCTAGCTGC | TTACCAACACCAGGCGATCC |
| *ACT11* | TGCATACGTTGGTGATGAGG | AGCCTTGGGGTTAAGAGGAG |

**Table S2**. Basic information and physical and chemical properties of *PvALDHs*

| **Family** | **Gene name** | **Gene id** | **chr** | **Location** | **Protein length** | **pI** | **Molecular weight** | **Instability index** | **Aliphatic index** | **Hydropathicity** | **Location** | **Conserved domain/sites** | | |
| --- | --- | --- | --- | --- | --- | --- | --- | --- | --- | --- | --- | --- | --- | --- |
|  |  |  |  |  |  |  |  |  |  |  |  | **PF00171** | **PS00687** | **PS00070** |
|  |  |  |  |  |  |  |  |  |  |  |  | **start-stop** | **yes/no** | **yes/no** |
| 2 | PvALDH2B1 | XP_007157468.1 | 2 | 10112472:10116406 | 538 | 6.58 | 58326.65 | 30.68 | 88.14 | -0.08 | Mitochondrion | 66-528 | yes | yes |
|  | PvALDH2B2 | XP_007153639.1 | 3 | 6528311:6532161 | 535 | 6.81 | 58402.79 | 30.21 | 88.47 | -0.098 | Mitochondrion | 63-525 | yes | yes |
|  | PvALDH2B3 | XP_007155410.1 | 3 | 41206815:41211557 | 535 | 7.66 | 58558.05 | 31.68 | 90.28 | -0.051 | Mitochondrion | 63-525 | yes | yes |
|  | PvALDH2B4 | XP_007151102.1 | 4 | 1854275:1858363 | 538 | 7.88 | 58556.18 | 40.18 | 90.15 | -0.009 | Mitochondrion | 57-511 | yes | yes |
|  | PvALDH2C1 | XP_007160345.1 | 2 | 47469524:47474952 | 501 | 5.88 | 54427.34 | 27.2 | 89.58 | -0.086 | Chloroplast | 32-491 | yes | yes |
|  | PvALDH2C2 | XP_007160347.1 | 2 | 47477556:47480195 | 450 | 6.14 | 48938.29 | 36.81 | 86.98 | -0.06 | Chloroplast. Cytoplasm | 1-440 | yes | no |
|  | PvALDH2C3 | XP_007152817.1 | 4 | 44427945:44433648 | 502 | 6.54 | 54596.93 | 28.16 | 90.96 | -0.001 | Chloroplast | 33-492 | yes | yes |
|  | PvALDH2C4 | XP_007152818.1 | 4 | 44444006:44447858 | 503 | 5.55 | 54718.03 | 32.72 | 91.93 | 0.055 | Chloroplast | 31-493 | yes | yes |
| 3 | PvALDH3F1 | XP_007156099.1 | 3 | 48678087:48682327 | 489 | 7.61 | 54734.84 | 42.46 | 100.65 | 0.004 | Chloroplast | 6-439 | no | no |
|  | PvALDH3H1 | XP_007161479.1 | 1 | 9737465:9743912 | 493 | 8.97 | 53491.05 | 34.12 | 103.06 | 0.041 | Chloroplast | 13-450 | yes | no |
|  | PvALDH3H2 | XP_007137428.1 | 9 | 18694801:18699010 | 490 | 6.44 | 53627.87 | 36.29 | 103.02 | -0.03 | Chloroplast | 13-447 | yes | no |
|  | PvALDH3I1 | XP_007159875.1 | 2 | 43932682:43939592 | 566 | 6.19 | 62746.63 | 42.99 | 97.84 | -0.004 | Chloroplast | 92-521 | no | no |
|  | PvALDH3J1 | XP_007150367.1 | 5 | 37531083:37534056 | 490 | 8.96 | 54420.39 | 45.5 | 100.86 | 0 | Chloroplast | 26-440 | no | no |
|  | PvALDH3J2 | XP_007134053.1 | 10 | 2494203:2501091 | 697 | 6.18 | 75342.2 | 36.16 | 106.05 | -0.029 | Cytoplasm | 15-438 | no | no |
| 5 | PvALDH5F1 | XP_007149178.1 | 5 | 5501199:5515494 | 347 | 5.23 | 36660.03 | 31.25 | 92.45 | 0.103 | Mitochondrion | 5-339 | yes | yes |
|  | PvALDH5F2 | XP_007149181.1 | 5 | 5550470:5554824 | 478 | 9.28 | 23629.45 | 45.01 | 91.14 | -0.104 | Mitochondrion | 68-435 | yes | yes |
| 6 | PvALDH6B1 | XP_007159167.1 | 2 | 37677604:37685270 | 537 | 8.51 | 57687.27 | 38.68 | 88.81 | -0.035 | Mitochondrion | 51-515 | no | yes |
| 7 | PvALDH7B1 | XP_007138652.1 | 9 | 33506902:33512327 | 424 | 5.28 | 45453.47 | 30.7 | 101.6 | 0.153 | Chloroplast | 33-417 | yes | no |
| 10 | PvALDH10A1 | XP_007155385.1 | 3 | 40942058:40945923 | 473 | 5.38 | 50768.64 | 26.5 | 96.81 | 0.095 | Chloroplast. Mitochondrion. Peroxisome | 21-472 | yes | yes |
|  | PvALDH10A2 | XP_007138125.1 | 9 | 26859474:26864172 | 503 | 5.15 | 54681.74 | 32.62 | 96.6 | -0.04 | Chloroplast. Mitochondrion. Peroxisome | 23-485 | yes | yes |
| 11 | PvALDH11A1 | XP_007161139.1 | 1 | 4843260:4847924 | 496 | 8.3 | 53045.42 | 34.09 | 91.43 | -0.002 | Cytoplasm | 24-483 | yes | yes |
|  | PvALDH11A2 | XP_007154898.1 | 3 | 36266403:36270902 | 497 | 6.76 | 53229.67 | 35.15 | 93 | 0.033 | Cytoplasm | 25-484 | yes | yes |
| 12 | PvALDH12A1 | XP_007155331.1 | 3 | 40457712:40465204 | 553 | 6.36 | 61360.47 | 35.92 | 89.35 | -0.115 | Mitochondrion | 131-495 | no | yes |
| 18 | PvALDH18B1 | XP_007163470.1 | 1 | 49717585:49723825 | 715 | 6.21 | 77767.4 | 33.8 | 109.13 | -0.045 | Cytoplasm | 291-558 | no | no |
|  | PvALDH18B2 | XP_007140469.1 | 8 | 14033763:14042292 | 720 | 6.11 | 78124.5 | 33.82 | 105.51 | -0.03 | Cytoplasm | 295-558 | no | no |
|  | PvALDH18B3 | XP_007141843.1 | 8 | 54503014:54510940 | 716 | 6.16 | 77805.27 | 33.87 | 108.97 | -0.025 | Cytoplasm | 287-559 | no | no |
| 22 | PvALDH22A1 | XP_007154371.1 | 3 | 28447193:28455391 | 597 | 7.55 | 65788.14 | 36.93 | 94.54 | 0.035 | Chloroplast | 60-527 | yes | yes |

Note: chr indicates the chromosome number and pI indicates the isoelectric point.

**Table S3.** Detailed information on the conserved motifs in the PvALDH proteins

| **No.** | **Motif** | **Width** | **Site** | **E-value** |
| --- | --- | --- | --- | --- |
| 1 | AYTLKEPJGVVGAITPWNFP | 20 | 24 | 8.6e-199 |
| 2 | FEDADJDKAVELALFAJFFNSGQICVAGSRVFVHESIYDEF | 41 | 16 | 1.2e-252 |
| 3 | BVKEDMRIAQEEIFGPVLPIIKFKTJEEAIKLA | 33 | 22 | 1.1e-234 |
| 4 | MFLWKVAPALAAGNTVVLKPSEQTPLSALYLAKL | 34 | 22 | 1.2e-257 |
| 5 | AAASNLKPVTLELGGKSPAIV | 21 | 26 | 8.4e-177 |
| 6 | PFGGYKMSGFGRYHGKYSLDKYLQVK | 26 | 17 | 3.4e-185 |
| 7 | EAGJPPGVLNVVTGFGPTAGAALASHMDVDKIAFTGSTDTG | 41 | 14 | 3.1e-234 |
| 8 | LAAGVFTKBIDTANRLSRAJRAGTVWINC | 29 | 23 | 1.5e-161 |
| 9 | GVZQGPQIDEEQFEKILSYIRSGKEEGATLLTGGKRVG | 38 | 13 | 8.2e-154 |
| 10 | PSAVRLFRYYAGAADKIHGLVVPADGDYH | 29 | 8 | 1.3e-089 |

**Table S4.** Expression of *PvALDHs* in different developmental stages and tissues

|  | **Flower buds** | **Flowers** | **Green mature pods** | **Leaves** | **Nodules** | **Root** | **Stem** | **Young pods** | **Young trifoliates** |
| --- | --- | --- | --- | --- | --- | --- | --- | --- | --- |
| PvALDH2B1 | 48.0903 | 67.6442 | 37.6253 | 5.57702 | 5.46589 | 8.07826 | 15.698 | 8.20824 | 9.29846 |
| PvALDH2B2 | 90.8612 | 151.213 | 101.571 | 52.9648 | 55.1421 | 139.932 | 79.6387 | 43.9191 | 36.5752 |
| PvALDH2B3 | 4.63353 | 31.5319 | 27.2541 | 53.6971 | 0.834737 | 1.09434 | 7.63103 | 8.95767 | 13.1088 |
| PvALDH2B4 | 0.471458 | 1.24079 | 23.9995 | 0.191003 | 0.382825 | 0.245325 | 2.1022 | 77.0575 | 0.841861 |
| PvALDH2C1 | 39.2162 | 101.938 | 47.8719 | 1.49122 | 72.016 | 166.675 | 7.01996 | 3.18448 | 0.527235 |
| PvALDH2C2 | 0.069033 | 0.034 | 0 | 0.058935 | 18.5859 | 37.6451 | 0.401838 | 0.081219 | 0.330041 |
| PvALDH2C3 | 23.1514 | 37.3425 | 5.09863 | 17.158 | 19.9701 | 32.9542 | 36.045 | 21.8612 | 9.95441 |
| PvALDH2C4 | 1.75141 | 1.38321 | 1.74167 | 0.166807 | 1.2457 | 0.602857 | 2.28992 | 0.278113 | 1.78585 |
| PvALDH3F1 | 66.4993 | 16.2176 | 1.91102 | 11.8645 | 0.41185 | 1.37682 | 2.54053 | 1.3918 | 7.35096 |
| PvALDH3H1 | 44.2742 | 575.616 | 135.785 | 48.2205 | 27.1429 | 57.9006 | 46.3271 | 54.3088 | 27.4255 |
| PvALDH3H2 | 3.78971 | 2.44942 | 119.686 | 1.29144 | 2.86688 | 1.98161 | 18.6257 | 5.58378 | 4.20383 |
| PvALDH3I1 | 7.10126 | 10.83 | 9.66154 | 17.7299 | 0.0271104 | 0.0410647 | 4.02501 | 14.1129 | 16.0184 |
| PvALDH3J1 | 100.819 | 32.9068 | 70.2885 | 46.6367 | 65.9092 | 57.2694 | 49.6662 | 55.189 | 53.0195 |
| PvALDH3J2 | 27.8394 | 6.56366 | 13.2472 | 36.6935 | 0.140489 | 0.505825 | 13.1581 | 2.14362 | 53.3734 |
| PvALDH5F1 | 42.9909 | 29.0532 | 30.3078 | 20.8763 | 14.8569 | 15.5157 | 15.5121 | 25.7976 | 18.1279 |
| PvALDH5F2 | 42.9909 | 29.0532 | 30.3078 | 20.8763 | 14.8569 | 15.5157 | 15.5121 | 25.7976 | 18.1279 |
| PvALDH6B1 | 42.9501 | 53.8421 | 57.0137 | 16.9831 | 40.559 | 80.0282 | 24.5657 | 49.8727 | 21.39 |
| PvALDH7B1 | 323.628 | 240.469 | 96.1441 | 17.0817 | 32.3304 | 117.092 | 74.8461 | 82.4951 | 14.3973 |
| PvALDH10A1 | 100.819 | 32.9068 | 70.2885 | 46.6367 | 65.9092 | 57.2694 | 49.6662 | 55.189 | 53.0195 |
| PvALDH10A2 | 150.89 | 250.44 | 25.1556 | 17.9518 | 12.3397 | 23.8406 | 22.5088 | 67.2919 | 34.0963 |
| PvALDH11A1 | 0 | 0.01544 | 0.341002 | 0.127961 | 0.121769 | 0.500754 | 0.108679 | 0.114356 | 0.291187 |
| PvALDH11A2 | 29.8496 | 23.0581 | 51.1 | 64.1588 | 0.058231 | 0.260892 | 38.1577 | 60.979 | 146.67 |
| PvALDH12A1 | 17.1287 | 14.7207 | 18.5883 | 5.90384 | 21.5513 | 19.2385 | 13.1929 | 30.7261 | 3.91611 |
| PvALDH18B1 | 10.7213 | 3.98741 | 3.0575 | 3.62205 | 11.0847 | 11.7812 | 2.16185 | 6.14891 | 11.0785 |
| PvALDH18B2 | 0.535543 | 0.038116 | 0.127676 | 0.54651 | 0.124026 | 0.239411 | 0.741395 | 0.311267 | 0.395451 |
| PvALDH18B3 | 7.44902 | 4.83913 | 12.5878 | 11.1035 | 15.5496 | 13.0199 | 8.71588 | 10.4094 | 11.1361 |
| PvALDH22A1 | 15.7028 | 7.47506 | 15.4173 | 29.0276 | 8.71465 | 14.0398 | 14.4838 | 14.5864 | 16.6708 |

**Table S5.** Expression pattern of *PvALDHs* under NS and AS stresses

| NS | Root 0h | Root 24h | Root 48h | Root 72h | Leaf 0h | Leaf 24h |
| --- | --- | --- | --- | --- | --- | --- |
| PvALDH2B2 | 1.00±0.00bc | 5.17±0.11a | 5.07±0.39a | 4.89±0.81a | 1.00±0.00bc | 1.10±0.37bc |
| PvALDH2C3 | 1.00±0.00c | 1.60±0.59c | 3.59±0.04b | 7.11±0.13a | 1.00±0.00c | 0.81±0.09c |
| PvALDH3H1 | 1.00±0.00c | 1.44±0.25b | 1.97±0.07a | 2.29±0.21a | 1.00±0.00c | 0.85±0.31c |
| PvALDH3H2 | 1.00±0.00c | 1.76±0.11ab | 2.02±0.39a | 1.50±0.27b | 1.00±0.00c | 0.48±0.02d |
| PvALDH5F1 | 1.00±0.00c | 2.41±0.60a | 2.82±0.33a | 2.69±0.26a | 1.00±0.00c | 1.50±0.45b |
| PvALDH6B1 | 1.00±0.00bc | 0.64±0.04cd | 0.64±0.02cd | 0.09±0.00d | 1.00±0.00bc | 1.67±0.35ab |
| PvALDH10A1 | 1.00±0.00c | 4.54±0.41a | 4.96±0.71a | 5.37±0.50a | 1.00±0.00c | 2.59±0.43b |
| PvALDH10A2 | 1.00±0.00d | 0.84±0.08d | 1.50±0.19cd | 2.31±0.04b | 1.00±0.00d | 1.24±0.43d |
| PvALDH22A1 | 1.00±0.00d | 2.52±0.36b | 2.08±0.16b | 6.71±0.16a | 1.00±0.00cd | 0.61±0.41d |
|  |  |  |  |  |  |  |
| AS | Root 0h | Root 24h | Root 48h | Root 72h | Leaf 0h | Leaf 24h |
| PvALDH2B2 | 1.00±0.00c | 1.12±0.44c | 1.11±0.06c | 1.06±0.16c | 1.00±0.00c | 1.98±0.02b |
| PvALDH2C3 | 1.00±0.00cd | 1.78±0.39c | 3.66±0.11b | 4.76±0.07a | 1.00±0.00cd | 0.43±0.06d |
| PvALDH3H1 | 1.00±0.00c | 0.21±0.03d | 0.44±0.04d | 1.27±0.36bc | 1.00±0.00c | 2.30±0.35a |
| PvALDH3H2 | 1.00±0.00d | 2.06±0.43ab | 1.08±0.11d | 1.58±0.36abc | 1.00±0.00d | 1.55±0.34ab |
| PvALDH5F1 | 1.00±0.00c | 1.57±0.09bc | 3.17±0.05a | 2.63±0.22b | 1.00±0.00c | 1.36±0.18bc |
| PvALDH6B1 | 1.00±0.00c | 1.32±0.56bc | 4.05±0.33a | 1.76±0.41b | 1.00±0.00c | 1.77±0.06b |
| PvALDH10A1 | 1.00±0.00cd | 1.70±0.17bc | 2.37±0.41ab | 1.82±0.36b | 1.00±0.00cd | 0.23±0.04d |
| PvALDH10A2 | 1.00±0.00c | 0.85±0.11d | 1.00±0.10c | 1.40±0.13b | 1.00±0.00c | 1.92±0.09a |
| PvALDH22A1 | 1.00±0.00c | 0.31±0.09d | 0.46±0.07d | 1.01±0.10c | 1.00±0.00c | 1.19±0.16c |

**Table S6.** Prediction of the secondary structure of the identified PvALDH proteins

| **No** | **Protein** | **No Protein Alpha helix (Hh)** | **Extended Strand (Ee)** | **Beta Turn (Tt)** | **Random Coil (Cc)** |
| --- | --- | --- | --- | --- | --- |
| 1 | PvALDH2B1 | 42.01% | 16.54% | 8.92% | 32.53% |
| 2 | PvALDH2B2 | 43.55% | 15.51% | 7.29% | 33.64% |
| 3 | PvALDH2B3 | 43.93% | 16.26% | 7.66% | 32.15% |
| 4 | PvALDH2B4 | 45.40% | 16.28% | 7.66% | 30.65% |
| 5 | PvALDH2C1 | 40.32% | 19.16% | 8.78% | 31.74% |
| 6 | PvALDH2C2 | 44.22% | 16.89% | 7.56% | 31.33% |
| 7 | PvALDH2C3 | 38.92% | 18.36% | 7.78% | 34.93% |
| 8 | PvALDH2C4 | 40.36% | 16.90% | 7.75% | 34.99% |
| 9 | PvALDH3F1 | 45.19% | 15.54% | 7.36% | 31.90% |
| 10 | PvALDH3H1 | 39.76% | 17.85% | 7.51% | 34.89% |
| 11 | PvALDH3H2 | 43.06% | 16.12% | 6.73% | 34.08% |
| 12 | PvALDH3I1 | 42.05% | 17.14% | 6.71% | 34.10% |
| 13 | PvALDH3J1 | 45.10% | 16.33% | 6.94% | 31.63% |
| 14 | PvALDH3J2 | 46.54% | 15.24% | 7.72% | 30.49% |
| 15 | PvALDH5F1 | 40.63% | 18.73% | 8.07% | 32.56% |
| 16 | PvALDH5F2 | 46.44% | 18.20% | 6.49% | 28.87% |
| 17 | PvALDH6B1 | 36.69% | 17.69% | 6.70% | 38.92% |
| 18 | PvALDH7B1 | 44.10% | 16.27% | 5.66% | 33.96% |
| 19 | PvALDH10A1 | 43.13% | 15.43% | 7.82% | 33.62% |
| 20 | PvALDH10A2 | 42.35% | 16.10% | 7.95% | 33.60% |
| 21 | PvALDH11A1 | 40.32% | 18.35% | 6.65% | 34.68% |
| 22 | PvALDH11A2 | 42.66% | 18.51% | 7.24% | 31.59% |
| 23 | PvALDH112A1 | 39.24% | 15.01% | 4.70% | 41.05% |
| 24 | PvALDH18B1 | 48.67% | 17.76% | 6.57% | 26.99% |
| 25 | PvALDH18B2 | 47.64% | 17.92% | 5.97% | 28.47% |
| 26 | PvALDH18B3 | 49.16% | 19.13% | 6.42% | 25.28% |
| 27 | PvALDH22A1 | 44.39% | 13.40% | 6.70% | 35.51% |

**Table S7.** Prediction of the N-glycosylation sites of all the identified PvALDH proteins

| **No.** | **Name** | **No of site** | **Position** | **Region** | **Score** |
| --- | --- | --- | --- | --- | --- |
| 1 | PvALDH2B1 | 0 | — | — | — |
| 2 | PvALDH2B2 | 0 | — | — | — |
| 3 | PvALDH2B3 | 2 | 398 | NATL | 0.5976 |
|  |  |  | 455 | NRTR | 0.5998 |
| 4 | PvALDH2B4 | 1 | 442 | NATS | 0.5886 |
| 5 | PvALDH2C1 | 0 | — | — | — |
| 6 | PvALDH2C2 | 0 | — | — | — |
| 7 | PvALDH2C3 | 0 | — | — | — |
| 8 | PvALDH2C4 | 0 | — | — | — |
| 9 | PvALDH3F1 | 4 | 75 | NKSL | 0.7528 |
|  |  |  | 206 | NLTP | 0.1833 |
|  |  |  | 227 | NPSE | 0.5572 |
|  |  |  | 400 | NDTM | 0.4406 |
| 10 | PvALDH3H1 | 0 | — | — | — |
| 11 | PvALDH3H2 | 0 | — | — | — |
| 12 | PvALDH3I1 | 0 | — | — | — |
| 13 | PvALDH3J1 | 1 | 382 | NKTL | 0.5897 |
| 14 | PvALDH3J2 | 1 | 380 | NKTL | 0.5581 |
| 15 | PvALDH5F1 | 0 | — | — | — |
| 16 | PvALDH5F2 | 0 | — | — | — |
| 17 | PvALDH6B1 | 2 | 125 | NVTT | 0.808 |
|  |  |  | 165 | NVSH | 0.5233 |
| 18 | PvALDH7B1 | 3 | 5 | NNSF | 0.588 |
|  |  |  | 40 | NPSN | 0.6156 |
|  |  |  | 44 | NQTI | 0.6312 |
| 19 | PvALDH10A1 | 3 | 29 | NPST | 0.5073 |
|  |  |  | 415 | NDTV | 0.4578 |
|  |  |  | 448 | NCSQ | 0.6158 |
| 20 | PvALDH10A2 | 0 | — | — | — |
| 21 | PvALDH11A1 | 3 | 25 | NKSS | 0.7669 |
|  |  |  | 37 | NPST | 0.474 |
|  |  |  | 413 | NASN | 0.4273 |
| 22 | PvALDH11A2 | 2 | 38 | NPTT | 0.6022 |
|  |  |  | 414 | NASN | 0.4686 |
| 23 | PvALDH12A1 | 3 | 16 | NHSG | 0.45 |
|  |  |  | 345 | NWSK | 0.5908 |
|  |  |  | 489 | NGTT | 0.5077 |
| 24 | PvALDH18B1 | 4 | 77 | NSSF | 0.5772 |
|  |  |  | 176 | NDSL | 0.6441 |
|  |  |  | 563 | NIIV | 0.6279 |
|  |  |  | 651 | NAST | 0.4231 |
| 25 | PvALDH18B2 | 3 | 74 | NSSF | 0.5801 |
|  |  |  | 179 | NDSL | 0.621 |
|  |  |  | 654 | NAST | 0.3957 |
| 26 | PvALDH18B3 | 2 | 177 | NDSL | 0.644 |
|  |  |  | 652 | NAST | 0.3949 |
| 27 | PvALDH22A1 | 4 | 424 | NHSM | 0.3769 |
|  |  |  | 455 | NDSK | 0.5271 |
|  |  |  | 469 | NQSR | 0.4309 |
|  |  |  | 586 | NSSG | 0.3796 |
